# A Metagenomics Transect into the Deepest Point of the Baltic Sea Reveals Clear Stratification of Microbial Functional Capacities

**DOI:** 10.1371/journal.pone.0074983

**Published:** 2013-09-23

**Authors:** Petter Thureborn, Daniel Lundin, Josefin Plathan, Anthony M. Poole, Britt-Marie Sjöberg, Sara Sjöling

**Affiliations:** 1 School of Natural Sciences and Environmental Studies, Södertörn University, Huddinge, Sweden; 2 Department of Molecular Biology and Functional Genomics, Stockholm University, Stockholm, Sweden; 3 Science for Life Laboratories, Royal Institute of Technology, Solna, Sweden; 4 Department of Biochemistry and Biophysics, Stockholm University, Stockholm, Sweden; 5 School of Biological Sciences, University of Canterbury, Christchurch, New Zealand; Argonne National Laboratory, United States of America

## Abstract

The Baltic Sea is characterized by hyposaline surface waters, hypoxic and anoxic deep waters and sediments. These conditions, which in turn lead to a steep oxygen gradient, are particularly evident at Landsort Deep in the Baltic Proper. Given these substantial differences in environmental parameters at Landsort Deep, we performed a metagenomic census spanning surface to sediment to establish whether the microbial communities at this site are as stratified as the physical environment. We report strong stratification across a depth transect for both functional capacity and taxonomic affiliation, with functional capacity corresponding most closely to key environmental parameters of oxygen, salinity and temperature. We report similarities in functional capacity between the hypoxic community and hadal zone communities, underscoring the substantial degree of eutrophication in the Baltic Proper. Reconstruction of the nitrogen cycle at Landsort deep shows potential for syntrophy between archaeal ammonium oxidizers and bacterial denitrification at anoxic depths, while anaerobic ammonium oxidation genes are absent, despite substantial ammonium levels below the chemocline. Our census also reveals enrichment in genetic prerequisites for a copiotrophic lifestyle and resistance mechanisms reflecting adaptation to prevalent eutrophic conditions and the accumulation of environmental pollutants resulting from ongoing anthropogenic pressures in the Baltic Sea.

## Introduction

The Baltic Sea is the world’s second largest body of brackish water, sustained by inflow of freshwater from the surrounding drainage area and only occasional inflow of oxygen-rich saltwater from the North Sea via the Danish straits [1]. In the Baltic Proper (i.e. the central Baltic Sea including the Western and Eastern Gotland Basins) these inflows create strong oxygen and saline gradients from surface to bottom, with stagnant water below the halocline [2], [3]. The Baltic Sea thus exhibits extreme stratification, with denser water below the halocline (60–80 m) being prevented from vertical mixing. Deeper waters thus become hypoxic or anoxic as oxygen is consumed through heterotrophic respiration during mineralisation of deposited organic matter [4]. As a result, some of the world’s largest marine ‘dead zones’ exacerbated by anthropogenic activity exist in the Baltic Proper [5].

Stratification of microbial community structure and functional capacity has been observed in a range of marine environments [6], [7], [8], suggesting links between community taxa, functional capacity and environmental parameters. The steep halocline in the Baltic Proper, together with considerable eutrophication, leads to extreme differences in environment, such that very different microbial processes may dominate above and below the halocline. A number of studies have provided insights on the taxonomic diversity of microbial communities both in the water column [9], [10], [11], [12], [13] and coastal sediment [14], [15] while the study of functional capacity has primarily focused on specific functions [16], [17], [18], [19], [20], [21], [22], [23], [24] and the study of total community function in the Baltic Proper is therefore still in its infancy.

Hypoxia is another defining feature of the Baltic Sea, and has been a periodic feature since its formation [25], [26]. However, hypoxia induced by human activity has become more widespread and prevalent in modern times [27]. Over the past decade, eutrophication has led to substantial spring and summer phytoplankton blooms, resulting in extensive anoxia and hypoxia in the deep waters and sediment of the Baltic Proper [25], [28], [29], [30]. Consequently, the oxygen gradient at Landsort Deep is among the steepest and most persistent. As such, it may provide unique information on the microbial ecosystem at steep environmental stratification. At depth, oxygenation events are rare in the Baltic Proper, the most recent events below the halocline occurring in the mid-1990s and the beginning of 2003 [31].

The Baltic Sea has a large catchment, and a further major environmental problem, anthropogenic pollution, is well documented. Long-lived pollutants, such as heavy metals and polyaromatic hydrocarbons (PAHs), are known to accumulate in sediments. While levels of some organic pollutants such as DDT are reducing [32], recent monitoring surveys at Landsort Deep show that concentrations of cadmium, copper, chromium, arsenic, lead, zinc and several persistent organic pollutants have increased in the sediment in the 21^st^ century [32].

We sought to establish the degree to which microbial communities with their functional capacities are stratified in the Baltic Proper. Our chosen study site was Landsort Deep, which, as the deepest point in the Baltic Sea, is minimally affected by occasional inflows, leading to sustained stratification, with acute anoxia below the halocline [31]. The wide range of environmental conditions at Landsort Deep, spanning hyposaline surface waters through anoxic sediment, and considerable accumulation of heavy metals, make this site an ideal one for examining the degree to which Baltic microbial communities are stratified. We sequenced water column metagenomes from three depths, and from anoxic sediment to establish whether the communities spanning the water column and the sediment were taxonomically distinct from one another, and whether each exhibits distinct functional capacity. We report strong stratification in both taxa and functional capacity, with the latter corresponding most closely to environmental parameters. The specifics of the stratification of taxa and predicted gene functions in relation to ecosystem functioning were analysed. Moreover, given that brackish water bacterial communities in the Baltic Sea appear to diverge in taxonomic diversity from other ocean environments [10], we also compared Landsort Deep metagenomes to other samples obtained elsewhere, including from open ocean environments and anoxic habitats, to examine whether Landsort Deep communities displayed unique features in taxonomic affiliation and/or functional capacity. The use of network-based analyses to compare Landsort Deep communities with those in other environments, also supports the uniqueness of microbial communities at Landsort Deep, further underscoring strong community stratification at this site. Finally, close examination of functional capacity reveals that the microbial community at Landsort Deep has unequivocally been shaped by both pollution and eutrophication.

## Results and Discussion

### Correspondence Analysis Reveals Substantial Community Stratification

**Figure 1:**
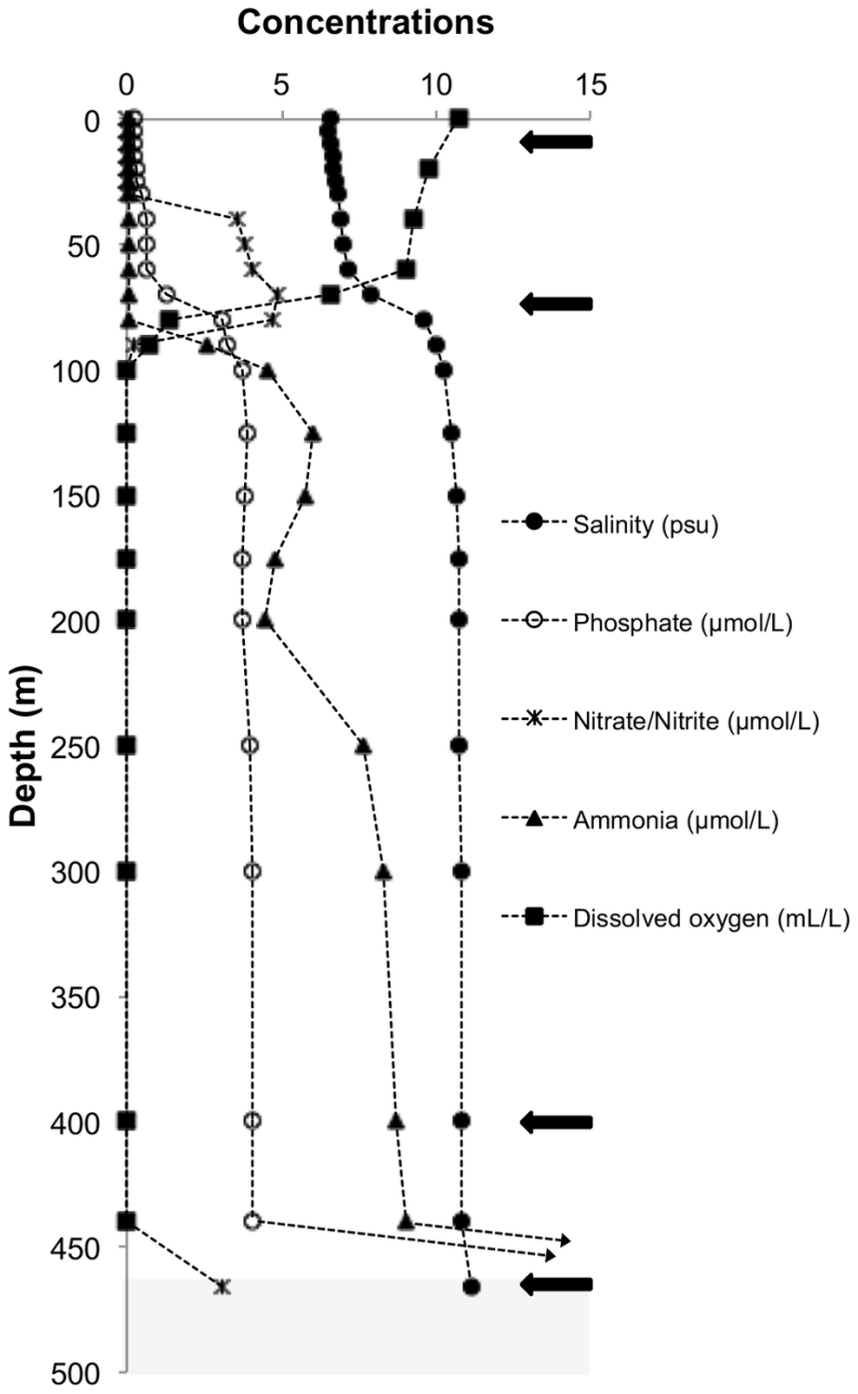
Environmental parameters of the Landsort Deep. Selected environmental parameters collected at Landsort, station BY31, Deep in the Baltic Sea in April 2009 concurrently with sampling of water and sediment for metagenomic analysis. Projected lines indicate the steep increase (10 to 100-fold) of NH_4_^+^ and PO_4_^3−^ in the sediment. Arrows indicates the four sampling depths. For additional environmental data see Table S1.

Metagenomes were generated from a bottom sediment sample and three water column samples across a depth transect spanning Landsort Deep surface water (10–20 m), the mixed layer (70–80 m), anoxic water (400–410 m) and bottom sediment (466 m). The environments from which each of the four metagenome samples were derived showed marked differences in key environmental parameters, including salinity, temperature, light and concentrations of dissolved oxygen, nitrate, ammonium, sulphide and phosphorous. Of particular note is the pronounced oxygen stratification across the study transect from surface water through to the bottom sediment (Figure 1 and Table S1).

The metagenomes of the four microbial communities sampled generated approximately 417 Mb of 454 GS FLX Titanium sequencing data consisting of 1 205 630 sequence reads, with an average sequence length of 344 bp, after removal of duplicate sequence reads using CDHIT-454 (Table S2). For each dataset, approximately 40–50% of the sequence reads could be assigned to taxa and 20% assigned a putative function (based on assignment of reads to SEED categories) (Table S2). These proportions are not unusual for marine metagenomes [6].

The triplicate sediment samples showed similar taxonomic and functional profiles as shown by low Bray-Curtis distances (Table S3). Similarities between replicates indicate that the sequencing depth was enough to provide representative profiles of the microbial communities. With this background, the sediment triplicates were pooled in subsequent analyses.

**Figure 2:**
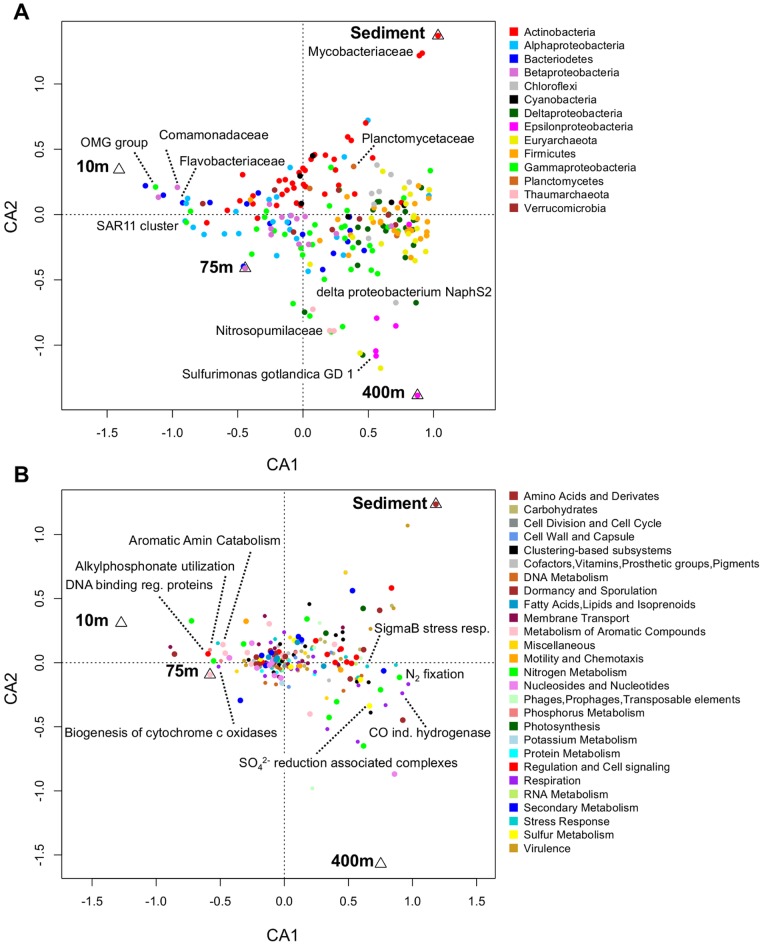
Correspondence analysis of taxa and functional capacities of Landsort Deep metagenomes. Correspondence analysis of the sampled Landsort Deep bacterial/archaeal communities based on taxa and functional capacity, respectively. a) Ordination diagram from CA of relative abundances of taxa (rank of family). CA1 and CA2 represent 59% and 27% of the total variation, respectively. Families affiliated to phylum (or class in the case of Proteobacteria) contributing with >1% to the total communities is shown. The most dominant family or species within each phylum/class is indicated with text if >0.9%. b) Ordination diagram from CA of relative abundances of functional capacities represented by SEED categories (hierarchy 2). Of the total variation 76% and 14% were reproduced on CA1 and CA2, respectively. SEED categories, within parent categories (SEED hierarchy 1) showing large variation (i.e. >25% difference between highest and lowest value), are shown with larger circles. The four SEED categories at the furthest ends of CA1 with relative abundance >0.1‰ in each community or >0.5‰ in at least one community, are shown. Triangles represent the positions of samples in the ordination diagram. For detailed data see Table S5.

Given that the four environments across the depth transect showed substantial differences in environmental parameters (Figure 1 and Table S1), we sought to establish to what extent this impacts community composition, and which environmental parameters most strongly contribute to sculpting the microbial communities. We therefore performed correspondence analysis (CA) on reads assigned to bacterial or archaeal taxonomic families (Figure 2a) and reads assigned to SEED categories (Figure 2b). In both analyses, the first ordination axis (CA1), representing the environmental parameters with largest influence, correlates best with dissolved oxygen, salinity and temperature (Pearson’s correlation coefficient, Table S4). Stratification was most evident for predicted gene function, where 76% (at SEED hierarchy 2) of the total variation corresponds to CA1 (Figure 2b). Substantial correspondence was also observed for community taxa, with 59% (at rank of family) of the total variation being accounted for by CA1 (Figure 2a).

### Metagenomic Reconstruction of Nitrogen and Sulphur Metabolisms at Landsort Deep

With evident microbial community stratification, we next examined how this was reflected in the N-dependent and the S-dependent functional capacities, respectively, across the Landsort Deep metagenomic transect. The Baltic Proper is characterised by excessive loading of nutrients and a ‘vicious circle’ of feedbacks that exacerbate eutrophication and drive phytoplankton blooms [25], [30], [33]. During the spring phytoplankton bloom, inorganic nitrogen bioavailable after the winter period and previous summer blooms, is consumed and depleted above the halocline, creating conditions for subsequent cyanobacterial summer blooms [34]. Below the halocline inorganic nitrogen, particularly ammonium, accumulates (together with phosphorous) from organic matter remineralisation and since aerobic nitrification can not proceed. However, dissimilatory nitrate reduction to ammonium (DNRA) and denitrification, important for nitrogen removal, may [35]. Heterotrophic denitrification, occurs under hypoxic conditions when NO_3_^−^ is available [36].

**Figure 3:**
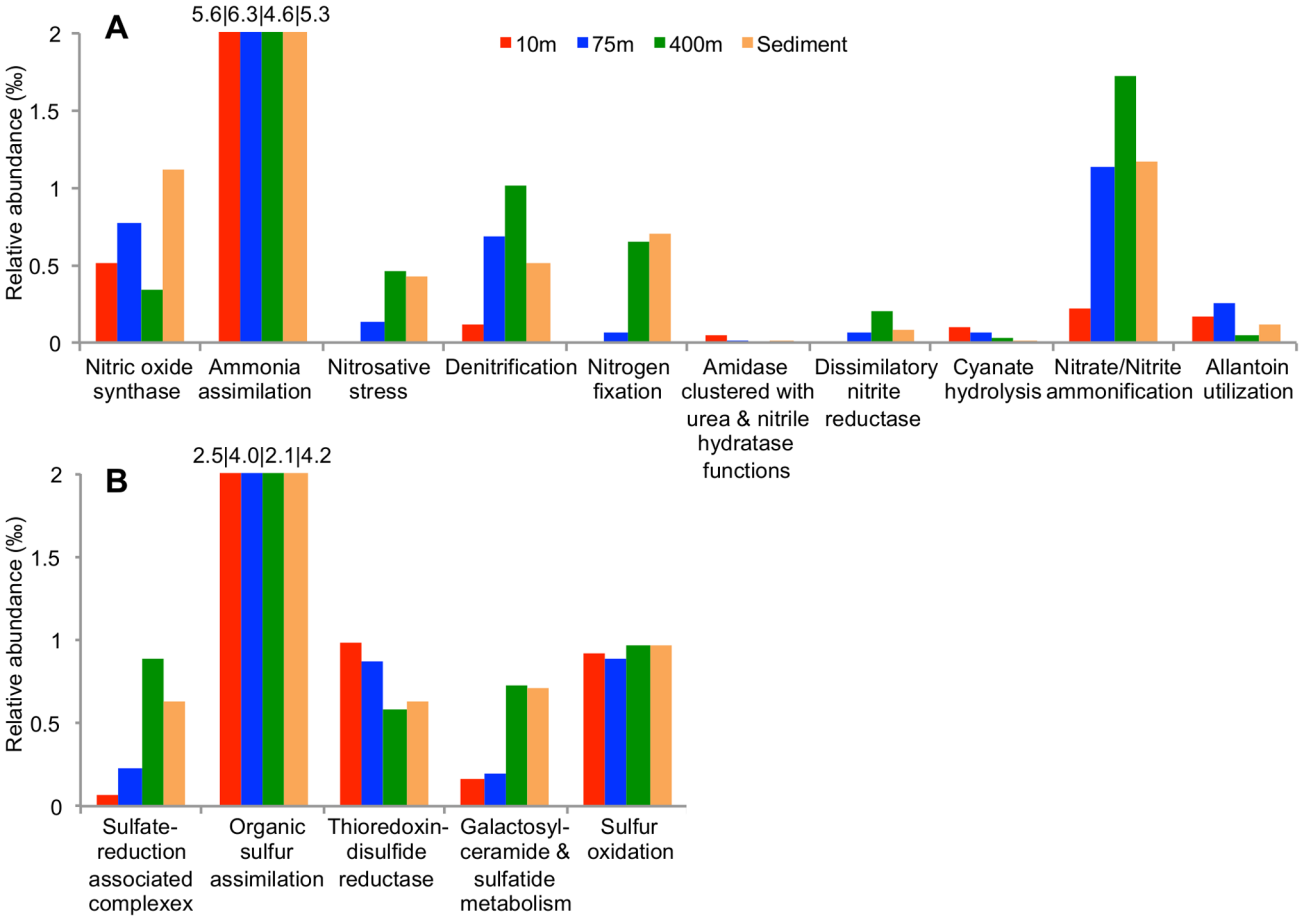
Nitrogen and sulphur metabolism of the Landsort Deep. Relative abundance of reads assigned to SEED categories within a) nitrogen metabolism and b) sulphur metabolism for the four Landsort Deep metagenomes. Numbers above truncated bars represent corresponding max value for the 10 m, 75 m, 400 m and sediment metagenome, respectively.

We report clear differences between communities in their capacity for denitrification and nitrate/nitrite ammonification (Figures 3a and S1). Interestingly, denitrification capacity was highest in the anoxic 400 m community (Figures 3a and S1), where NO_2_^−/^NO_3_^−^ concentrations were low (Figure 1). Such conditions typically reduce heterotrophic denitrification N-removal, since the process is dependent on regeneration of substrates (NO_2_^−^ and NO_3_^−^) through nitrification at oxic conditions [35], [36]. The high abundance of denitrification genes in deeper sulphidic waters at 400 m might thus be a result of chemolithotrophic denitrification [37], [38], [39]. Consistent with this, we observed abundant (0.9% of the total archaeal/bacterial community at species level) sequence reads classified as Epsilonproteobacterium *Sulfurimonas gotlandica* GD1 at 400 m (Figure 2a), that has the genetic capacity for chemolithotrophic denitrification coupled with sulphur oxidation [40]. Likewise, a large fraction (35%) of denitrification genes at 400 m derive from Epsilonproteobacteria, and sulphur oxidation capacity (Figure 3b) is also evident at anoxic depths where 18% of the total sulphur oxidation genes were assigned to Epsilonproteobacteria. Sulphur-oxidising Epsilonproteobacteria have been found to be an important catalyst of chemolithoautotrophic denitrification and dark CO_2_-fixation at Gotland Deep [17], [18]. The high abundance of these organisms in the anoxic water metagenome of Landsort Deep (Figure S2), also when compared to metagenomes from other oxygen limited environments (Figure S3, see unclassified Campylobacterales, Campylobacteraceae and Helicobacteraceae), implies an active role in nitrogen and sulphur transformation also at this site.

We find that the distribution of Epsilonproteobacteria largely coincides across the Landsort Deep transect (Pearson’s r = 0.83) with sulphide insensitive Thaumarchaeota [41], the latter being particularly abundant at 400 m (7.1%), but also at 75 m (6.2%) (Figure S2). The Thaumarchaeota reads appear closely related to *Nitrosopumilus maritimus* which oxidizes ammonium to nitrite [42]. Other studies of Landsort Deep and Gotland Deep have indicated an important role for archaeal ammonium oxidation at the suboxic depths in the Baltic Proper [22], [43]. The comparatively high abundance of Thaumarchaeota observed in our data was evident also in comparison to most other aquatic metagenomes (Figure S3, see Nitrosopumilaceae). The ammonia oxidizing archaea suggests a source of the substrate necessary for denitrification by the Epsilonproteobacteria. As well as a high abundance of Thaumarchaeota (Figure S2), we also report presence of archaeal *amo*ABC genes (annotated using IMG/M-ER) in the mixed layer and anoxic water at Landsort Deep. This co-distribution of sulphur-oxidizing epsilonproteobacteria and ammonium oxidizing Thaumarchaeota is indicative of a syntrophic interaction.

Over the past decade the concentration of ammonium, as a product of organic matter mineralization, has drastically increased below the halocline at Landsort Deep (The Baltic Nest Institute, http://nest.su.se/). We therefore assessed if the increased availability of ammonium coincides with nitrogen removal through anaerobic oxidation of ammonium (anammox). Anammox bacteria related reads (*Candidatus* Kuenenia stuttgartiensis) were identified primarily in the anoxic water (0.3%) and sediment (0.3%) but no hydrazine oxidase genes were detected. At the time of sampling we therefore find no capacity for anammox at Landsort Deep. While this may reflect finite sampling, we note that this result is consistent with another recent study where no anammox activity was reported for Landsort Deep in spring 2009, despite anammox being detected at the hypoxic/anoxic zone of Gotland Deep [38].

Nitrogen might also be prevented from being removed, and instead recycled or stored in the ecosystem at reduced conditions, potentially increasing the eutrophication effect through anaerobic dissimilatory reduction of nitrate to ammonia (Jänti and Hietanen, 2012). We detected only few *nrf*A assigned reads (2–26 fold less frequent compared to *nir*S at any suboxic or anoxic depth), indicating that a role for DNRA, at the time of sampling would be limited.

Fixation of nitrogen is at least as important process as nitrogen removal. We detected the minimum gene set required for nitrogen fixation (*nif*HDK and *nif*ENB) and found clear stratification of this process, at subsystem level, across Landsort Deep communities, with an increased capacity at depth (Figures 2b, 3a and S1). Several sequences also matched to *nif*A, *nif*U and *nif*S, but these belong to highly conserved superfamilies, and thus pose a misinterpretation risk [44], [45]. Capacity does not equal activity however, and high ambient DIN has generally been considered to inhibit nitrogen fixation. That said, benthic nitrogen fixation may well occur despite high ambient ammonium concentrations, as demonstrated more recently (for review see [46]). Sampling time, i.e. during the spring bloom that consists of phytoplankton other than cyanobacteria, plus the fact that filtering may serve to exclude filamentous diazotrophs, may together explain the low N fixation capacity observed at surface water, which in summer has abundant nitrogen fixating cyanobacteria. As the capacity for nitrogen fixation correlated strongly both with the capacity for sulphate reduction (r = 0.94) and abundance of Deltaproteobacteria (r = 0.95) across the Landsort Deep gradient (Figures 3 and S2), nitrogen fixation capacity in the anoxic water was supported by the fact that 36% of *nif*HDK and *nif*ENB were assigned to sulphate-reducing Deltaproteobacteria including the highly abundant (5.4% of the total community at species level) sulphate-reducing and naphthalene degrading Deltaproteobacteria NaphS2 [47]. Sulphate-reducing Deltaproteobacteria may therefore be key diazotrophs at the anoxic zone of Landsort Deep. These sulphate reducers were even more abundant than the Desulfobacteraceae (3.4%) at 400 m (Table S5). The results suggest that the sulphidic environment, which has gradually increased over the past decade in the Baltic Proper, may carry less studied links between nitrogen and sulphur metabolism, as revealed for anoxic minimum zones [39].

### Comparative Metagenomics Points to Environment as the Primary Influence on Community Composition and Functional Capacity at Landsort Deep

Analysis of the communities across the depth transect indicated that functional capacity and, to a lesser degree, community taxa are influenced by environment. Given that the deeper water of the Baltic Proper is largely cut off, with only irregular inflows of oxygenated marine water from the North Sea, this makes it very different from most other marine environments. We were therefore interested in establishing whether Landsort Deep microbial communities both show taxonomic makeup and functional capacities similar to microbial communities from similar environments, or whether isolation and extreme stability of the environment at Landsort Deep, particularly below the halocline, has resulted in unique communities with more similar genetic makeup than that of other communities. We therefore performed a large-scale comparative metagenomics analysis on both taxa and functional capacity spanning 11 different sites and 27 metagenome datasets (see methods), with samples derived from water column, sediment, soil and compost.

**Figure 4:**
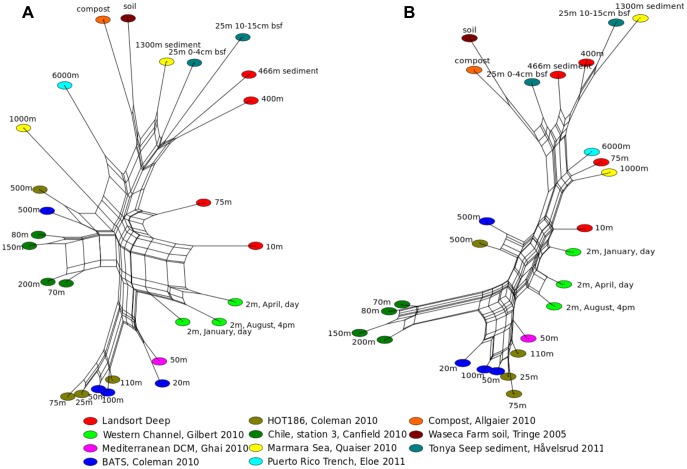
Comparative metagenomic analysis of taxa and functional capacities. Comparative network analysis of the Landsort Deep metagenomes in this study and other aquatic and terrestrial metagenomes of; (a) taxa (family level distribution); (b) functional capacity (SEED hierarchy 2). The network was obtained using the NeighborNet method and the Bray Curtis distance metric in MEGAN 4.

**Figure 5:**
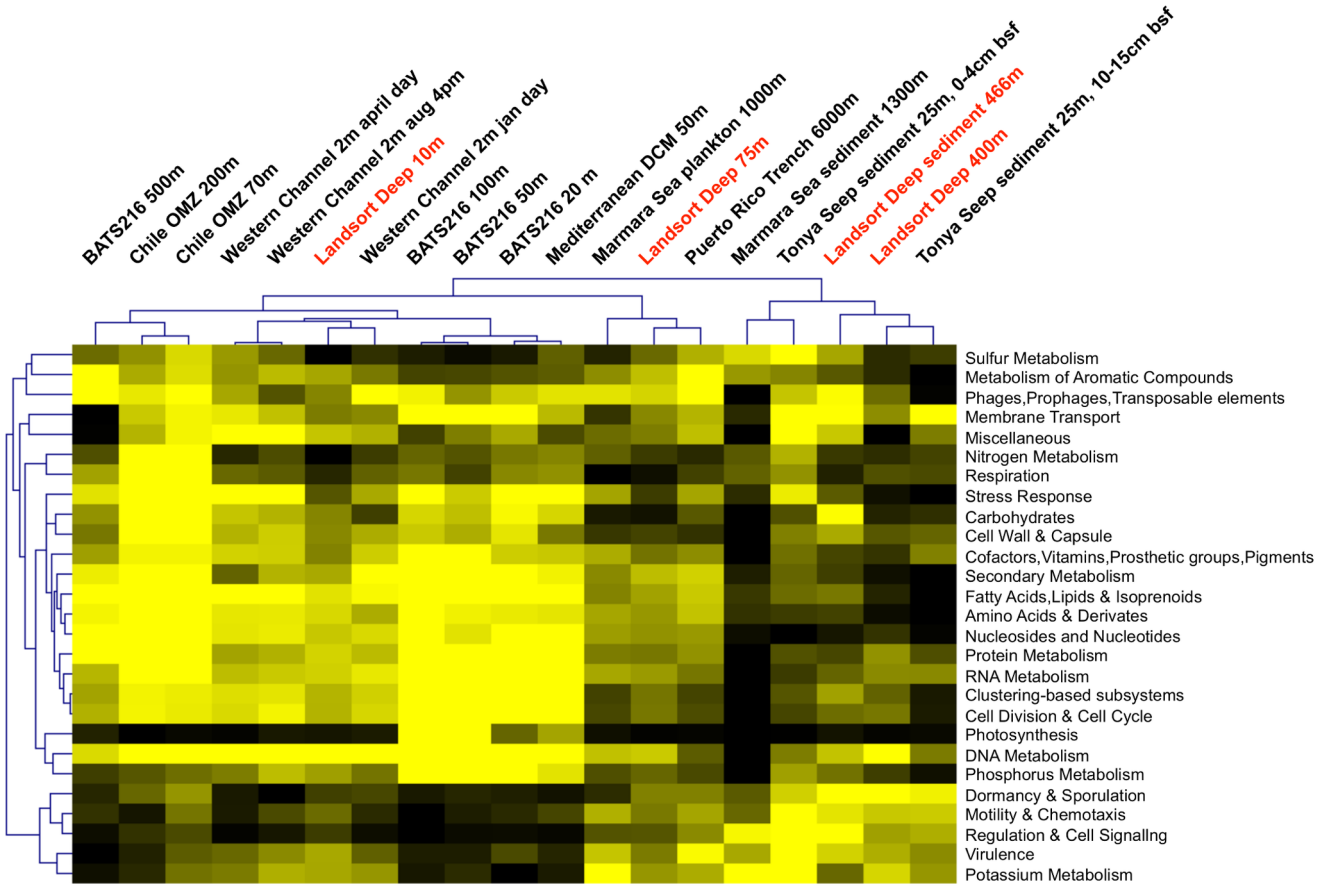
Comparative analysis and hierarchical clustering of functional capacities. Comparative analysis of the functional capacity of Landsort Deep metagenomes with that of other aquatic metagenomes based on normalised assignments of sequence reads to functional categories of SEED (hierarchy 1). Yellow to black represents high to low relative abundance of SEED categories. Clustering was generated using Kendall’s Tau distance metric and average linkage clustering. For a more detailed cluster analysis see Figure S4.

To generate a quantitative measure of similarity, we made use of phylogenetic network methods implemented in MEGAN 4 [48] to create distance metrics between all samples, both for inferred taxonomic affiliation and predicted gene function. As shown in Figure 4, both graphs reveal well-supported splits that group the anoxic Landsort Deep 400 m and sediment communities with other hypoxic and anoxic sediment communities by taxonomic affiliation (Figure 4a) and functional capacity (Figure 4b). The functional profiles in particular reveal strong similarities between sediment metagenomes from the Marmara Sea, Californian Tonya Seep and the deepest Landsort Deep microbial communities. Similarities grouping these metagenomes together, when compared to all other marine metagenomes, are significant (Welch’s t-test, Storey’s FDR q<0.05) enrichment in genes for regulation and cell signalling, motility and chemotaxis, and defence mechanisms (Figures 5 and S4) but underrepresentation in secondary- and fatty acid metabolism. Genes for enzymes that reflect the hypoxic/anoxic conditions (e.g. hydrogenases and anaerobic respiratory reductases) were significantly (q<0.05) enriched, and so were taxa of sulphate-reducing bacteria (e.g. Desulfobacterales and Desulfovibrionales) and methanogenic archaea (e.g. Methanosarcinales and Methanomicrobiales), consistent with shared environmental characteristics of these environments, i.e. strongly stratified environments with respect to oxygen, high pollution, anoxia and/or high nutrient and sedimentary load.

Highly similar taxonomic profiles are also evident between sediment and 400 m water column communities at Landsort Deep (Figures 4a and S3). This similarity is all the more striking given the differences in sample preparation i.e. filtering vs. non-filtering and DNA extraction, necessitated by the starting material, i.e. water and sediment. The most recent inflow of oxygen rich water into the Baltic Sea in mid-2003 led to oxygenation and flushing out of hydrogen sulphide at the Bornholm Basin and Gotland Deep, but had no effect on the deepest parts of Landsort Deep [28] thereby contributing to the isolation of these deep water communities.

The taxonomic profile of the surface water community at Landsort Deep groups closely with three surface water metagenomes from the Western English Channel. Similarities between these metagenomes include a significant (q<0.05) overrepresentation of Rhodobacteraceae, Sphingobacteriaceae, unclassified Methylophilales and unclassified Flavobacteria, the three latter potentially due to high loads of organic particles [49] and other coastally derived nutrients and riverine input [25], [50]. While there is a clear grouping on the basis of taxonomic affiliation (Figure 4a), the split based on functional capacity (Figure 4b) is less marked, though this may in part be a reflection of the very different levels of salinity between these two areas and the different sampling times for all four samples.

Of the Landsort Deep communities, the 75 m community, from the oxic-anoxic interface is an outlier when it comes to taxonomy, as it falls slightly outside the main groups that emerge in this analysis, the closest being surface water at Landsort Deep and Western Channel. The 75 m Landsort metagenome derives from a brackish environment with a steep biogeochemical gradient, so this community would not be expected to share similar taxonomical composition with open ocean communities of similar depths [51], [52]. In contrast to the unique taxonomical composition relative to other metagenomes examined in this analysis, it is striking that the functional capacity of the 75 m community is very similar to two metagenomes from considerably deeper communities, a trench off Puerto Rico at 6 km depth and at the Marmara Sea at 1 km depth (Figure 4b). Significant (q<0.05) similarities between these metagenomes are enrichment in the functional capacities belonging to DNA-binding and transcriptional regulation, archaeal replication, transcription and translation, motility (flagellum) and bacteriophages. We caution against overinterpretation here however, since there are no metagenomes in our dataset from closely comparable environments. Nevertheless, the similarity with such deep communities likely reflects the extent of eutrophication and the euxinic state at Landsort Deep, with limited water mixing, stagnant conditions and hypoxia shifting high up in the water column.

### Functional Capacities Characteristic of Landsort Deep Microbial Communities Reflect Anthropogenic Activity

Our CA results (Figure 2) indicate that functional capacity varies greatly across the Landsort Deep transect. We therefore examined which functional capacities underlie stratification. The anoxic communities (400 m and sediment) show comparatively high abundance in genetic prerequisites for attachment to and utilization of organic material, including chemotaxis, pilus formation, quorum sensing, biofilm formation, degradation of polysaccharides and amino sugars (Figures S1, S5 and Table S5). With a comparatively high abundance in motility, regulation and cell signalling and defence mechanism genes, and a comparatively low abundance of fatty acid and secondary metabolism genes (Figure 5), the functional capacities of Landsort Deep anoxic communities are consistent with a copiotrophic lifestyle [53], living on, for example, pico-pellets of sinking phytoplankton or chitin-rich exoskeleton remnants [54]. The comparatively high representation of these functional capacities most likely reflects the effect of organic material deposition and eutrophication.

Although functional capacity varies greatly across the Landsort Deep transect we observe that the metagenomes as a group, when compared against all other metagenomes, are significantly (Welch’s t-test, Storey’s FDR q<0.05) underrepresented in genes associated with biotin biosynthesis but enriched in functional capacities (SEED hierarchy 3) of osmoregulation and metabolite detoxification (glutathione-regulated potassium-efflux system protein KefC), transcriptional regulation (molybdate-binding domain of ModE) of several genes of biogeochemical pathway enzymes including dimethyl sulfoxide reductase, nitrate reductase and hydrogenases, and motility (flagellum), several possible indicators of a stratified environment.

Another probable indicator of eutrophication at Landsort Deep is the significant (Fisher’s exact test, Storey’s FDR, q<0.05) overrepresentation of Flavobacteriaceae (7.4%) (Figure S3) in surface waters. Flavobacteriaceae are common in coastal areas [55], including Western Channel surface waters [56], and are important for carbon transformation and hydrolysis of organic matter from diverse sources [49]. This group may therefore play a major role in transforming the carbon from spring and possibly summer blooms. Consistent with this, 33% of surface water reads assigned to genes involved in degradation of high molecular weight carbon (i.e. identified in the SEED categories Polysaccharides, Aminosugars and Glycoside hydrolase) could be matched to Flavobacteria.

Carbohydrate utilisation was also evident in sediment at Landsort Deep (Figure 5). Genes for degradation of cellulose (cell walls of plants) and chitin (e.g. exoskeletons of arthropods), identified in the same SEED categories as above, were primarily assigned to Actinobacteria (10–20%) and more specifically to Mycobacteria (3–8%). Notably, these were significantly (q<0.05) overrepresented (5-fold more abundant) in the Landsort Deep sediment compared to any of the other analysed metagenomes (Figure S3, see Mycobacteriaceae and Conexibacteraceae). We also detected mycobacterial sequences with best matches to PAH-degrading Mycobacteria (40% of all mycobacteria), which could have a role in PAH degradation at Landsort Deep. Although the sediments have high levels of sedimentary organic carbon (Table S1), it is not clear whether the Mycobacterial signature is the result of sedimentation from the oxygenated waters (most sequences were best matched to aerobic pathogenic Mycobacteria) or if the populations are autochthonous for the sediment. For aerobic PAH degradation by Mycobacteria [57], sedimentation seems most likely, though PAH degradation also occurs under sulphate-reducing conditions [58]. As noted above, naphthalene degradation, could well be performed by Deltaproteobacteria NaphS2 [47] at these depths.

The impact of pollution at Landsort Deep was clearly reflected by a comparatively high abundance of resistance genes for cobalt, cadmium and zinc in the 400 m and sediment communities (Figure S5 and S6a). This is not surprising as cadmium and zinc concentrations in Landsort Deep are known to be high [32]. Marmara Sea and Tonya Seep sediments and Puerto Rico Trench deep water likewise show high abundance of cadmium-, cobalt- and zinc resistance genes. Generally, this may reflect the biogeochemistry of these environments with adsorption of the metals to biogenic material, or the formation of sulphides, sedimentation and accumulation of particles onto the sediment [59]. In the Baltic Sea, this process leads over time to increased trace metal levels in the sediment, not the least in a trench with accumulation bottom, such as the Landsort Deep.

Another probable sign of adaptation to a polluted environment is the comparatively high abundance of integrons in all Landsort Deep metagenomes (Figure S6b), in particular at anoxic depths. The only other community exhibiting higher levels of integrons was the Tonya Seep sediment. Integrons, genetic elements that act as vectors for the mobilisation of genes, can, through integration, permit adaptation of bacteria to their environment through the receipt of new genes, including resistance genes [60]. Integrons have previously been shown to be present at elevated levels in aquatic microbial communities in industrially contaminated environments [61], [62]. Expansion of mobile genetic elements with depth has been observed with increasing water depth in other studies [7], [63], [64] and suggested to be a reflection of relaxed selection pressure from slower growth rate and/or smaller population sizes of deep sea communities [63]. That the high integron abundance in the Landsort Deep shows parallels to both deep water and polluted environments suggests that, in environments suffering from long-term pollution, integrons may successfully proliferate across microbial genomes, perhaps on account of their tendency to carry genes that may positively influence recipient survival in polluted environments.

### Concluding Remarks

The unique conditions of the Baltic Proper combined with infrequent turnover of deeper waters have led to strong stratification of the water column. Our survey at Landsort Deep, the deepest point in the Baltic Sea, is the first metagenomic survey of any deep (>400 m) water or sediment of the Baltic Sea. It clearly shows strong stratification of both microbial taxa and functional capacities across a depth transect spanning from surface water, through the oxycline, into the anoxic deep waters and sediment. Significantly, functional capacity corresponds more closely to environment than does taxonomic affiliation, suggesting that a range of taxa may be using very similar functional gene repertoires to adapt to the considerable environmental constraints present across our sample site. As predicted given known physical parameters, we find a marked effect of the coupled parameters salinity, temperature and oxygen content on the microbial community. The unique oceanographic characteristics of the Baltic Proper, the bathymetry and the impact this has on nutrient dynamics and the distribution of pollutants, is reflected in the Landsort Deep microbial communities that are characterized by gene repertoires shaped by both anthropogenic pollution and eutrophication. The present study clearly documents this influence on Baltic Sea microbial communities at Landsort Deep.

## Materials and Methods

### Ethics Statement

Sampling was conducted within the Stockholm Marine Research Centre (currently Stockholm University Baltic Sea Centre) at monitoring station BY31. The station is not a national park, private land or protected area. No specific permissions were required. The study did not involve endangered or protected species.

### Sampling Procedure

Water and sediment samples were collected on 15 April 2009 during the spring bloom at Landsort Deep (lat 583591 N, long 01814.26 E) in the Baltic Sea, Sweden. Triplicate sediment cores for DNA extraction and elemental analysis were retrieved from 466 m depth using a Kajak sampler. Concomitantly, salinity, dissolved oxygen concentration, temperature and pH were measured in the water phase as close to the sediment surface as possible. Pore water was extracted from the sediment top layer (0–10 cm) using Rhizon Soil Moisture Samplers (Rhizosphere Research Products, Wageningen, The Netherlands). Water (30 L) was sampled from three distinct zones, the surface water at 10–20 m, the mixed layer at 70–80 m and the anoxic zone at 400–410 m depth, using 5 L Niskin bottles. Samples were pre-filtered through 3 µm 47 mm polycarbonate filters (GE Healthcare, Uppsala, Sweden) onto 0.22 µm Sterivex GS filters (Millipore, USA) that were immediately immersed in lysis buffer. Filtrates and sediment samples were frozen on board, transferred on dry ice to the laboratory and stored at −80°C, until DNA extraction.

Nutrient data were determined and provided by the Department of Systems Ecology at Stockholm University.

### DNA Extraction and Sequencing

Total DNA was extracted from the sediment top layer (0–10 cm) in 200 mg aliquots, using the FastDNA® SPIN Kit for Soil (MP Biomedicals, Solon, USA) and total DNA of filters from water samples was extracted according to [65] with a few modifications, see Text S1. All samples (5 µg/sample) were barcoded, using Multiple Identifiers (MIDs), and sequenced in one full plate run with Roche’s 454 GS FLX Titanium pyrosequencing technology (454 Life Sciences, Branford, CT, USA) at the Centre for Metagenomic Sequence analysis (CMS) at the Royal Institute of Technology in Stockholm. Sequence data were deposited at the European Nucleotide Archive (ENA) with accession numbers ERR268106 (10 m), ERR268107 (75 m), ERR268108 (400 m) and ERR268109-11 (sediment).

### Functional and Taxonomic Annotation

Duplicate sequence reads that passed standard GS FLX quality criteria removed with CDHIT-454 [66] using default parameters. Subsequently, the raw metagenomic sequence reads were aligned to the NCBI non-redundant (NR) database using BLASTX [67]. Metagenomic sequence reads were assigned to taxa and SEED [68] and KEGG [69] functional categories using MEGAN 4 [70], [71], and were also annotated with IMG/M-ER [72]. Only reads assigned to bacteria and archaea were considered for downstream analyses. Abundance data normalised to 100 000 reads per dataset were used for comparisons of relative abundance in all downstream analyses.

### Correspondence Analyses

CA of taxa (rank of phylum, family, genus and species) and functional capacity (SEED hierarchies 1, 2 and 3), based on abundance data normalised to 100000 reads per dataset, were performed in R [73] using the R package Vegan [74]. Subsequently, environmental data was correlated to the CA axes using the resulting site scores (positions of sites on CA axes) and Pearson’s correlation coefficient. Calculations of Pearson’s correlation coefficient were made using Vegan.

### Global Comparative Metagenomic Analyses

Landsort Deep data was compared with other metagenomes including marine metagenomes from the Marmara Sea [75], the Western Channel [76], the Sargasso Sea BATS216 [51], Hawaiian Ocean HOT186 [51], Mediterranean Sea [77], Chilean Oxygen Minimum Zone [52], the Tonya Seep of Santa Barbara Channel [78] and a Puerto Rico trench [79], but also Waseca farm soil [80] and switchgrass compost [81]. Network analysis was performed, as implemented in MEGAN 4 [48] using the Bray-Curtis distance metric, on abundance data normalised to 100000 reads per dataset. Subsequently to standardisation of abundance data within the range 0–1, hierarchical clustering was performed in MeV 4.6 [82] using Kendall’s tau distance metric and average linkage clustering. Statistically significantly over or underrepresented taxa and functional capacities for individual metagenomes, or cluster of metagenomes, were identified using STAMP [83]. Two-sided Fisher’s exact test combined with Storey’s FDR (q<0.05) was used for pairwise comparison of metagenomes. Clusters of marine metagenomes were identified from network analysis and compared to all the other metagenomes in a two-group test using Welch’s t-test and Storey’s FDR (q<0.05).

## Supporting Information

Figure S1 **Pairwise statistical analyses (n = 6) of sequence reads assigned to SEED categories (hierarchy 2) in the four Landsort Deep metagenomes.** Only statistically significant differences (Fisher’s exact test, Storey’s FDR q<0.05), where the SEED categories are represented with >100 reads in at least one metagenome, are shown.
(PDF)

Figure S2 **Relative abundance of sequence reads assigned to phyla (or classes in the case of Proteobacteria) within the bacterial/archaeal communities.** Only taxa with >1% of the assigned sequence reads in any of the four Landsort Deep communities were included.
(PDF)

Figure S3 **Comparative analysis of the taxonomic distribution in Landsort Deep communities with that of other aquatic metagenomes based on relative abundance of taxa at the family rank of the NCBI taxonomy in MEGAN.** Hierarchical clustering was generated with Kendall’s Tau distance metric and average linkage clustering.
(PDF)

Figure S4 **Pairwise statistical comparison of functional capacities (SEED hierarchy 1) between cluster of metagenomes.** The cluster (defined by comparative network analysis) of metagenomes of Landsort Deep 400 m and sediment, Marmara Sea sediment and Tonya Seep sediments was compared to all other marine metagenomes using Welch’s t-test and Storey’s FDR (q<0.05) in STAMP.
(PDF)

Figure S5 **Comparative analysis of functional capacity of Landsort Deep communities with that of other aquatic metagenomes based on relative abundance of SEED categories (hierarchy 3).** Yellow to black colour represents high and low relative abundance of SEED categories. Hierarchical clustering was performed using Kendall’s Tau distance metric and average linkage clustering.
(PDF)

Figure S6 Relative abundance of sequence reads assigned to the SEED categories Cobalt zinc cadmium resistance (a) and Integrons (b), respectively, in marine metagenomes. Clustering of metagenomes is based on results of the comparative network analysis (Figure 4). Horizontal lines indicate mean value for the respective cluster.
(PDF)

Table S1 Environmental parameters collected at Station BY31, Landsort Deep, Baltic Sea at the 15^th^ of April concurrently with sampling of water and sediment for metagenomic analyses.
(PDF)

Table S2 Summary of sequence and annotation data for the four Landsort Deep metagenomic data sets. Taxonomical and functional assignments of sequence reads were performed with MEGAN 4.
(PDF)

Table S3 Ecological distances between the Landsort Deep metagenomes. Dissimilarity indices were calculated with Bray-Curtis distance metric at the taxonomic level of family.
(PDF)

Table S4 Correlation between environmental parameters and distribution of functional capacities and taxa, respectively. Pearson’s correlations were performed based on coordinates of SEED categories (hierarchy 2) and taxa (family rank) along the three ordination axes from correspondence analysis.
(PDF)

Table S5 Species scores (coordinates) from CA for the different taxa (at the rank of family) and SEED categories (hierarchy 2), respectively. Shown are also abundances in each sample normalised to 100 000 reads per dataset. Taxa and SEED categories are ordered from low to high CA1 coordinates.
(PDF)

Text S1 **Supporting material and methods.**
(PDF)
