# Supplementary figures and images for: A Metagenomics Transect into the Deepest Point of the Baltic Sea Reveals Clear Stratification of Microbial Functional Capacities
Source: PLoS One. 2013 Sep 23;8(9):e74983. doi: 10.1371/journal.pone.0074983 (PMC3781128; doi:10.1371/journal.pone.0074983)

10m 75m

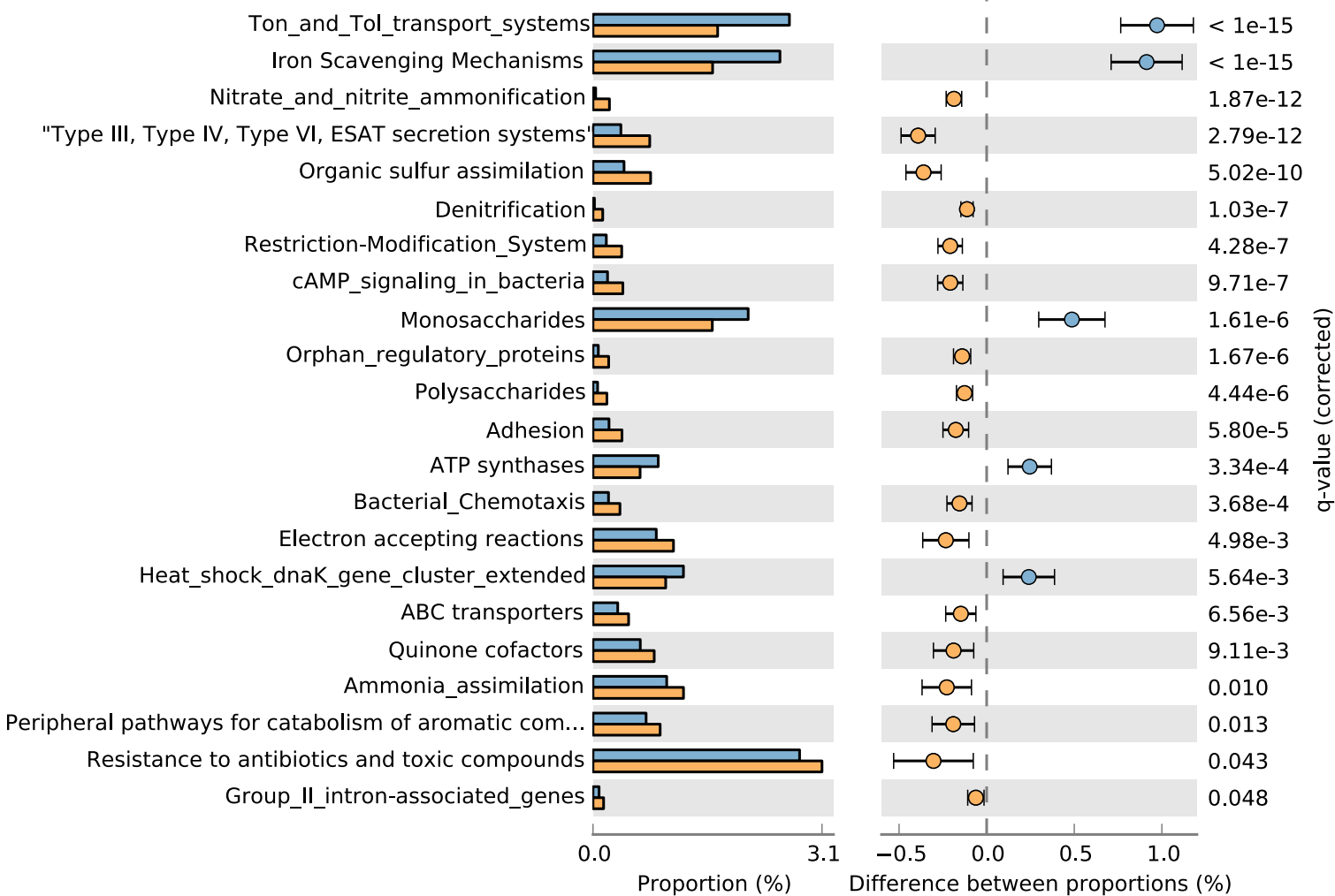

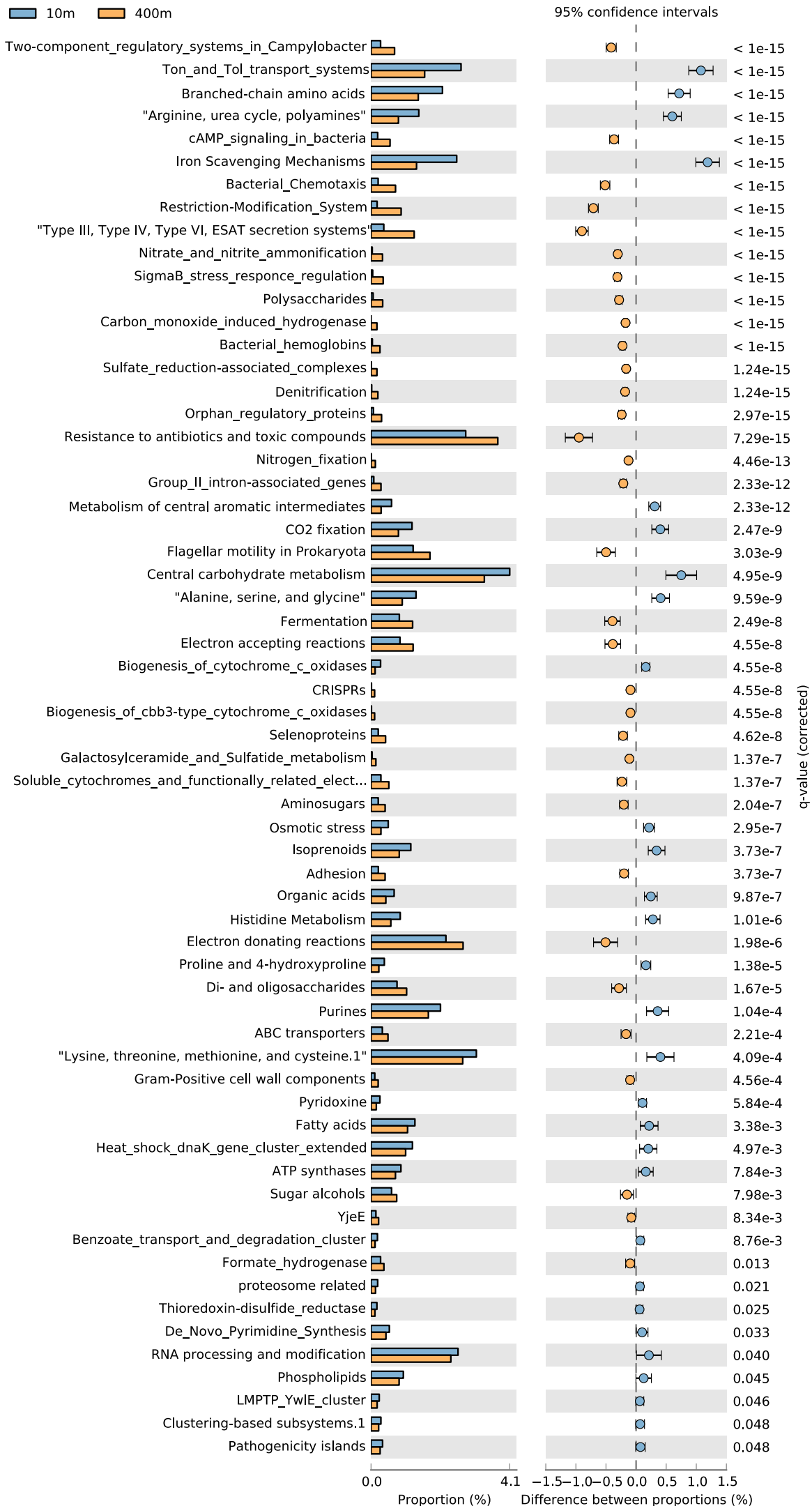

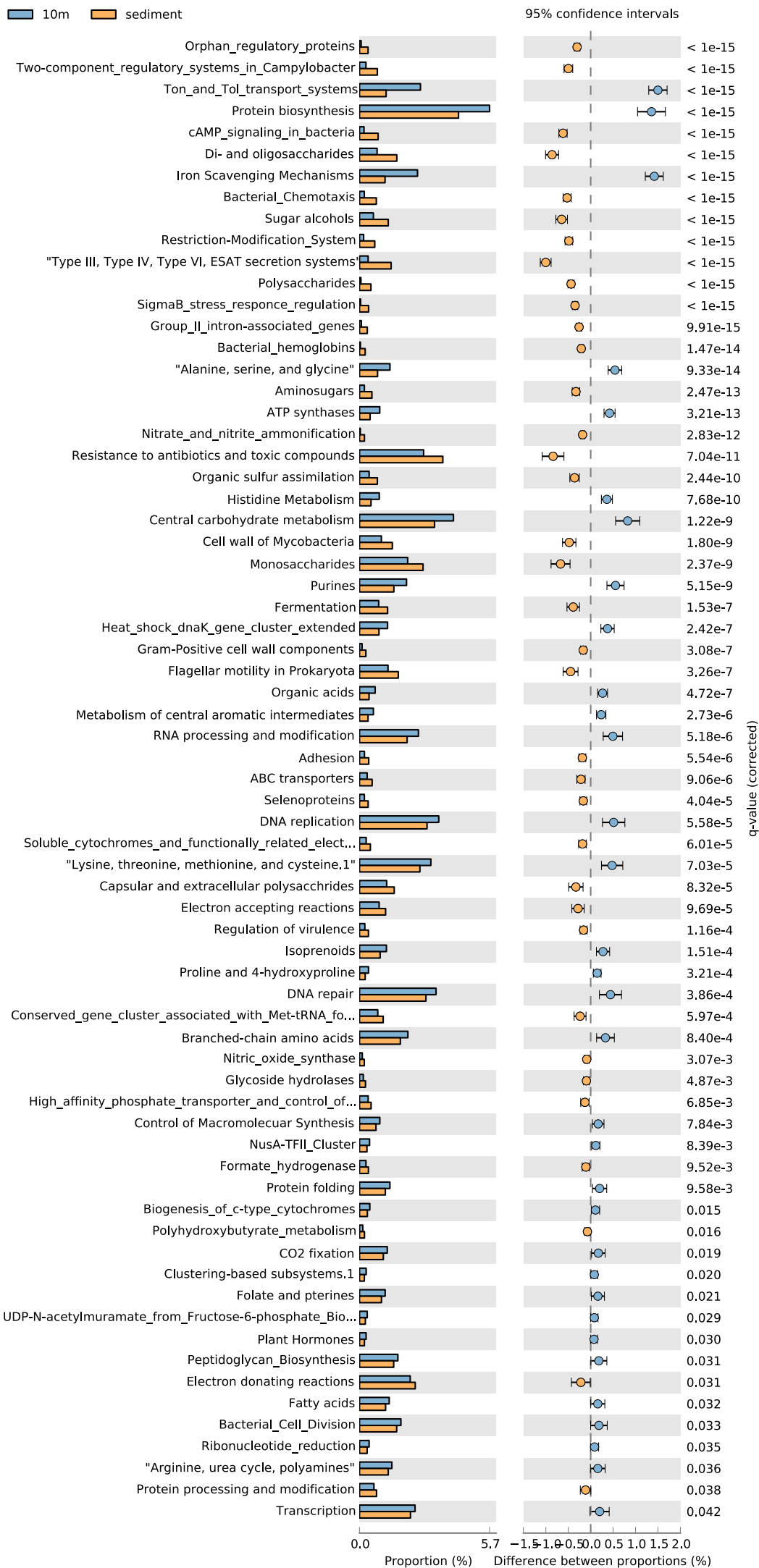

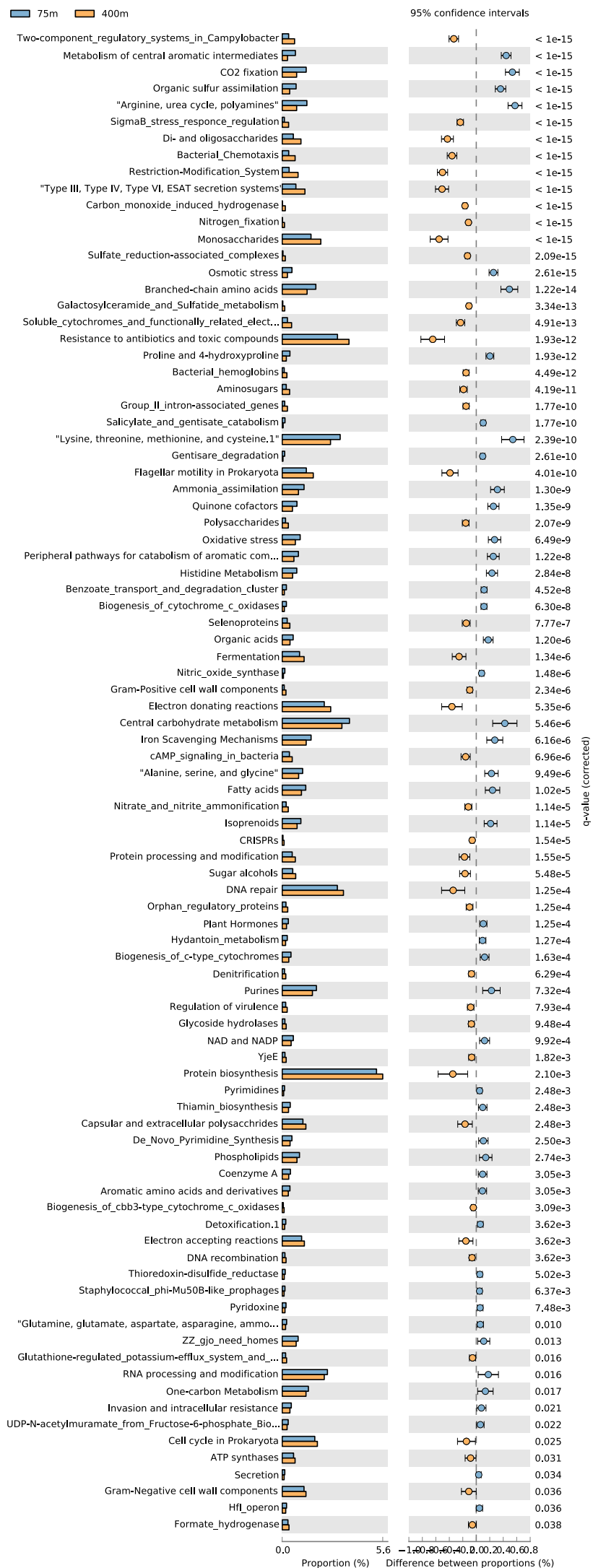

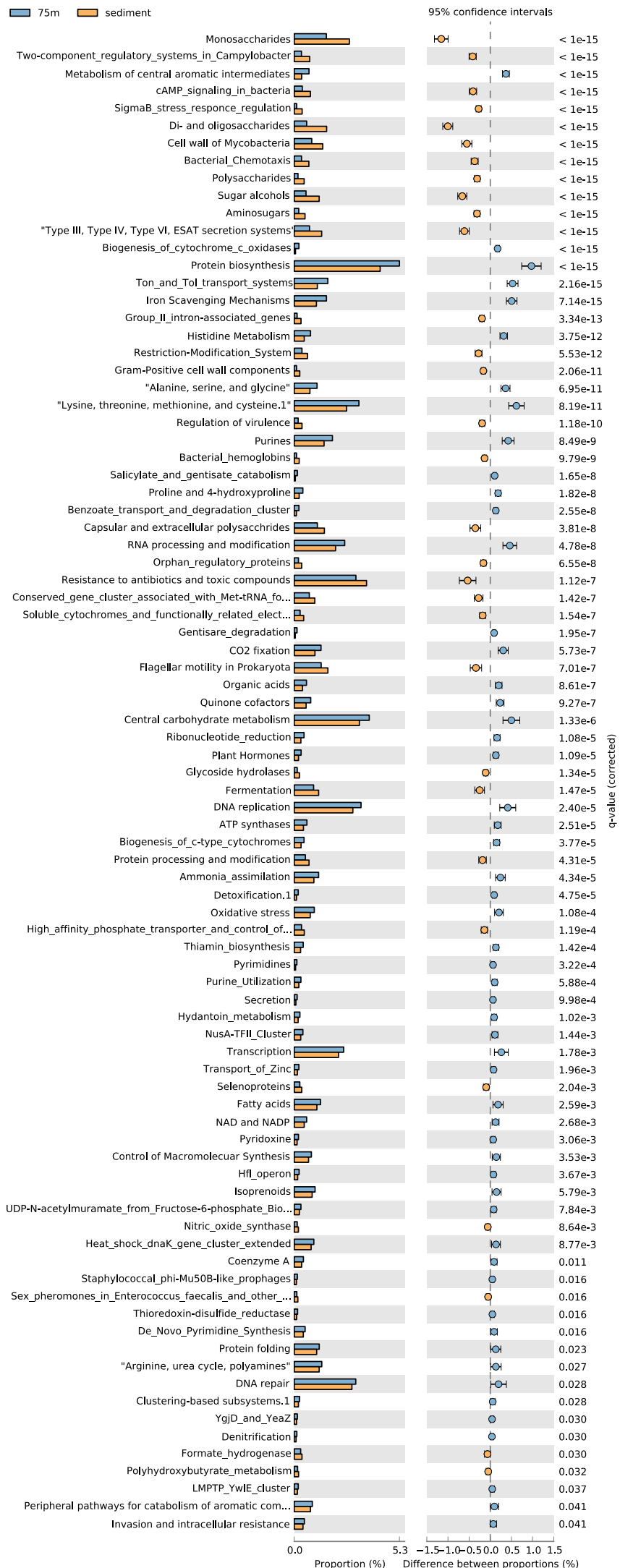

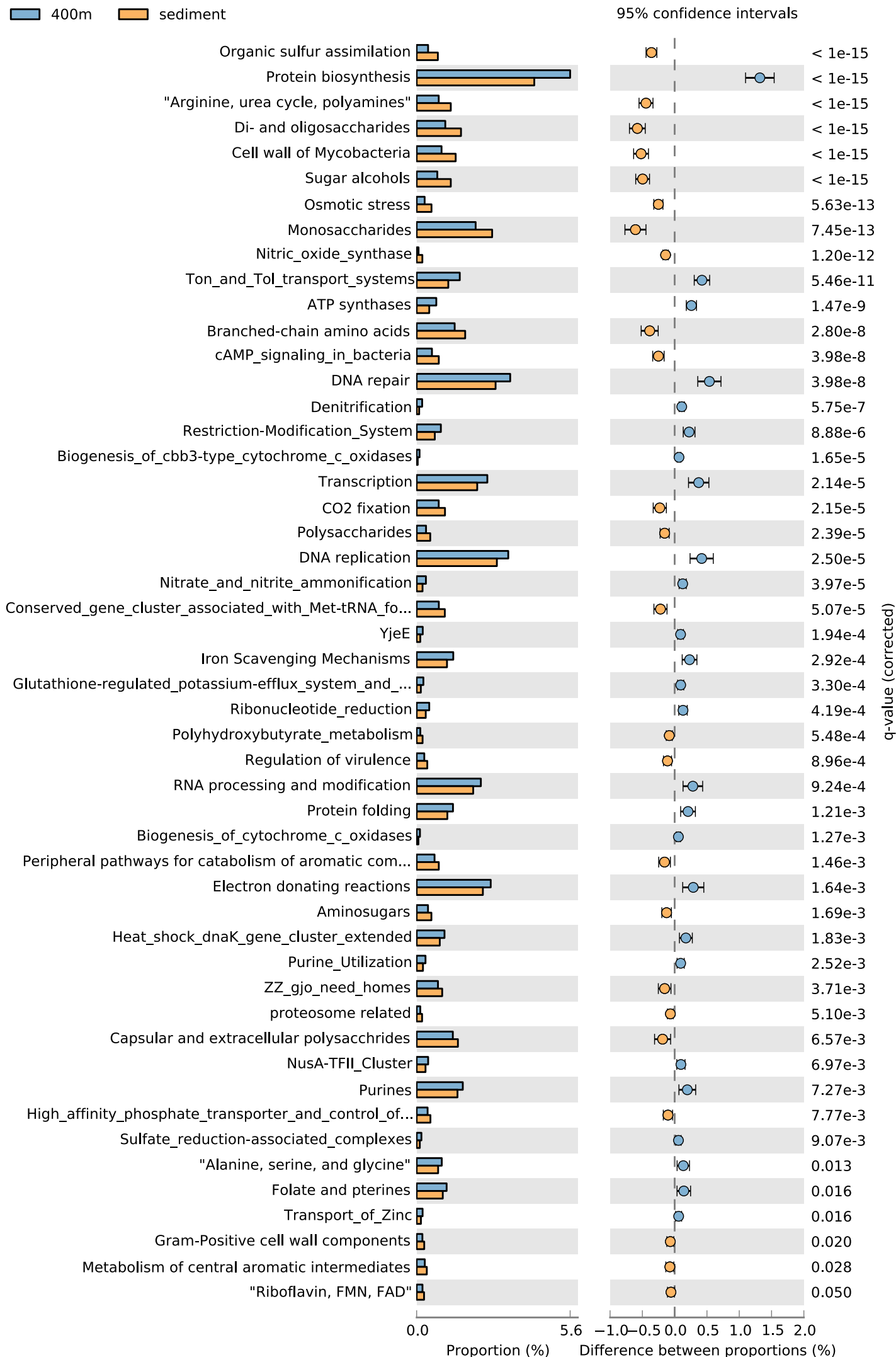

Supplement: Figure S1 — Pairwise statistical analyses (n = 6) of sequence reads assigned to SEED categories (hierarchy 2) in the four Landsort Deep metagenomes. Only statistically significant differences (Fisher’s exact test, Storey’s FDR q<0.05), where the SEED categories are represented with >100 reads in at least one metagenome, are shown. (PDF) [file pone.0074983.s001.pdf]

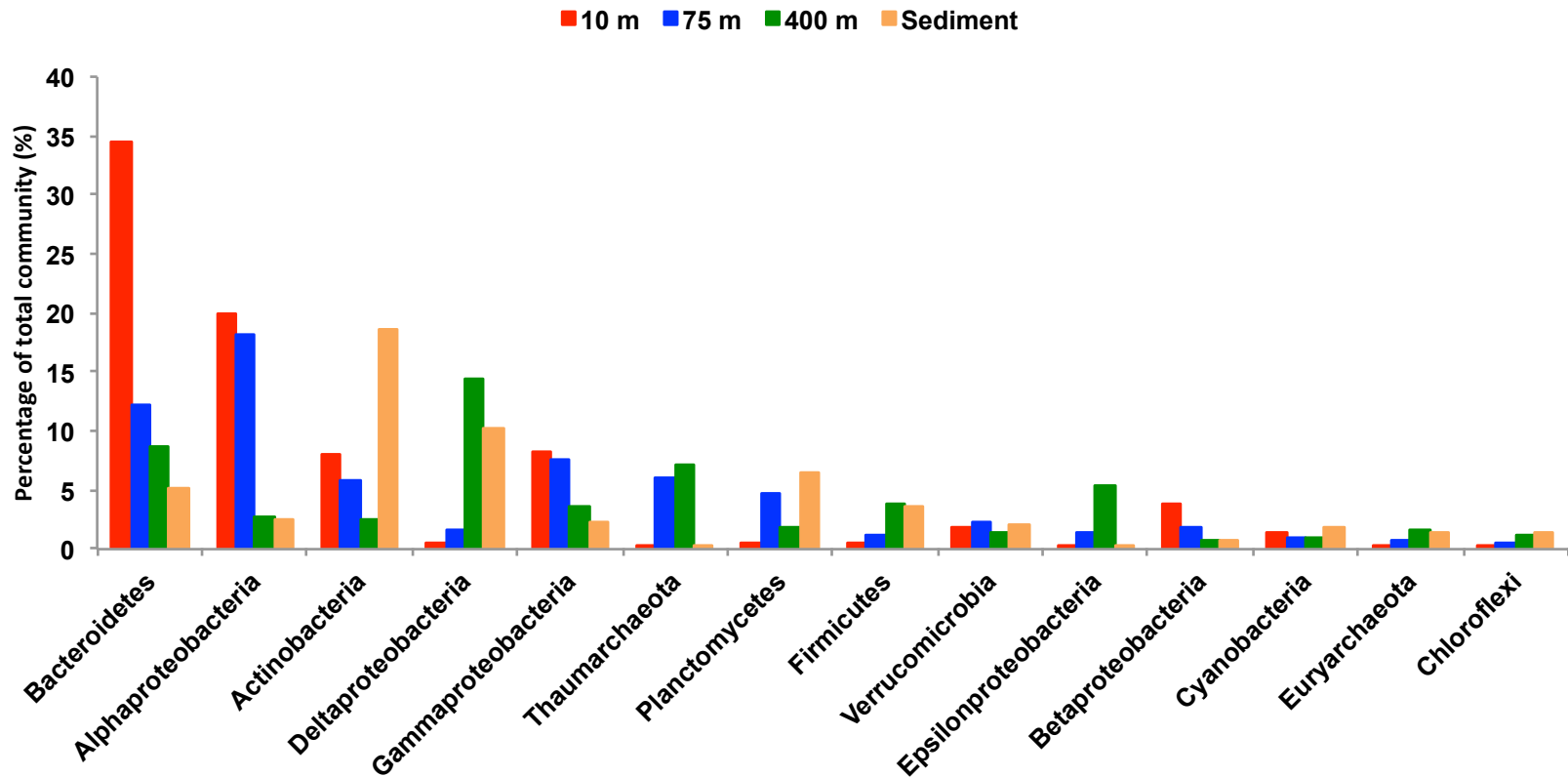

Supplement: Figure S2 — Relative abundance of sequence reads assigned to phyla (or classes in the case of Proteobacteria) within the bacterial/archaeal communities. Only taxa with >1% of the assigned sequence reads in any of the four Landsort Deep communities were included. (PDF) [file pone.0074983.s002.pdf]

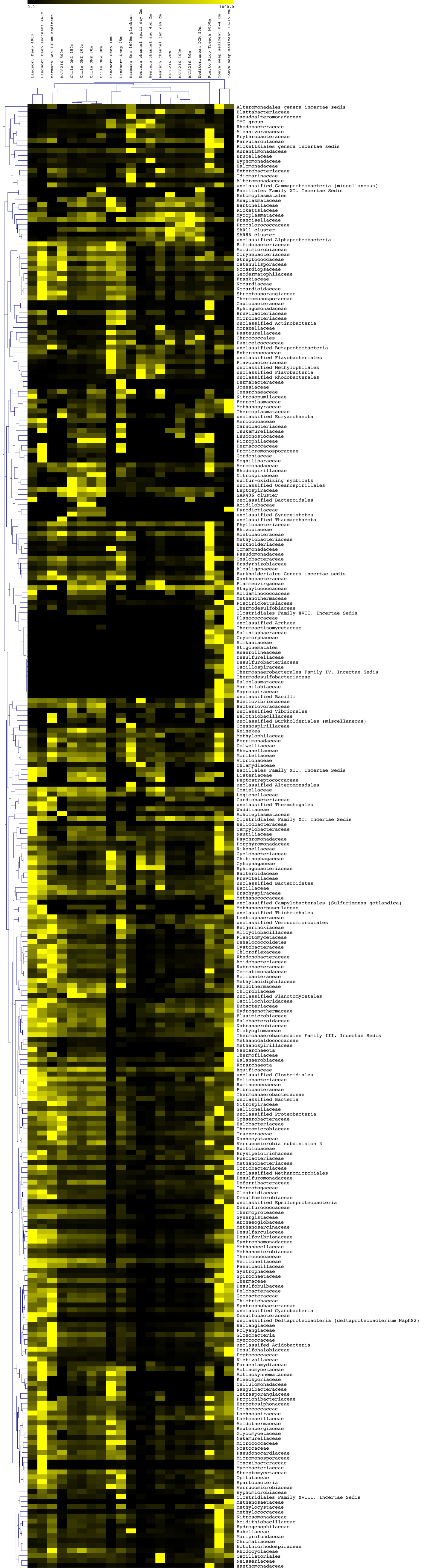

Supplement: Figure S3 — Comparative analysis of the taxonomic distribution in Landsort Deep communities with that of other aquatic metagenomes based on relative abundance of taxa at the family rank of the NCBI taxonomy in MEGAN. Hierarchical clustering was generated with Kendall’s Tau distance metric and average linkage clustering. (PDF) [file pone.0074983.s003.pdf]

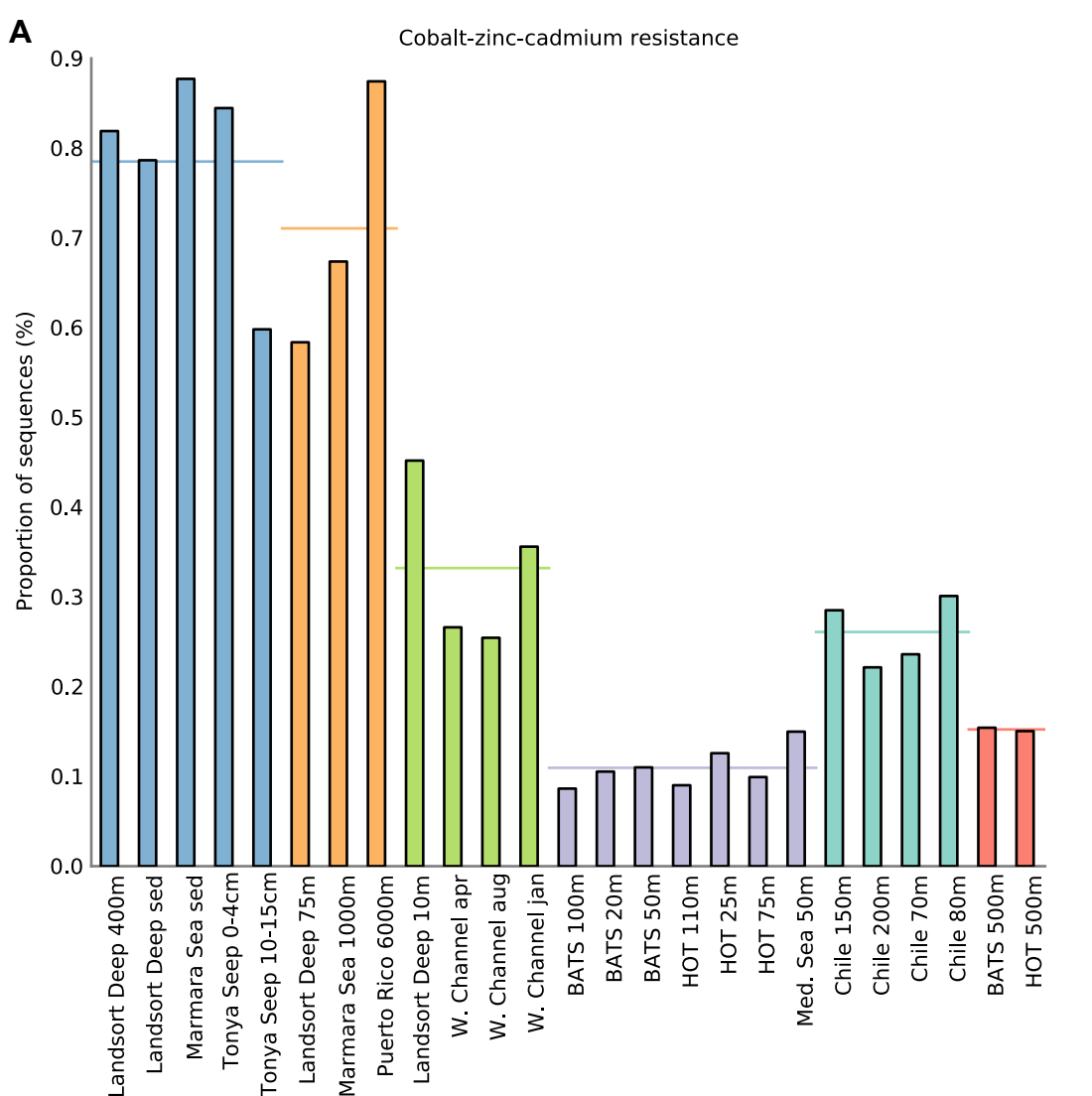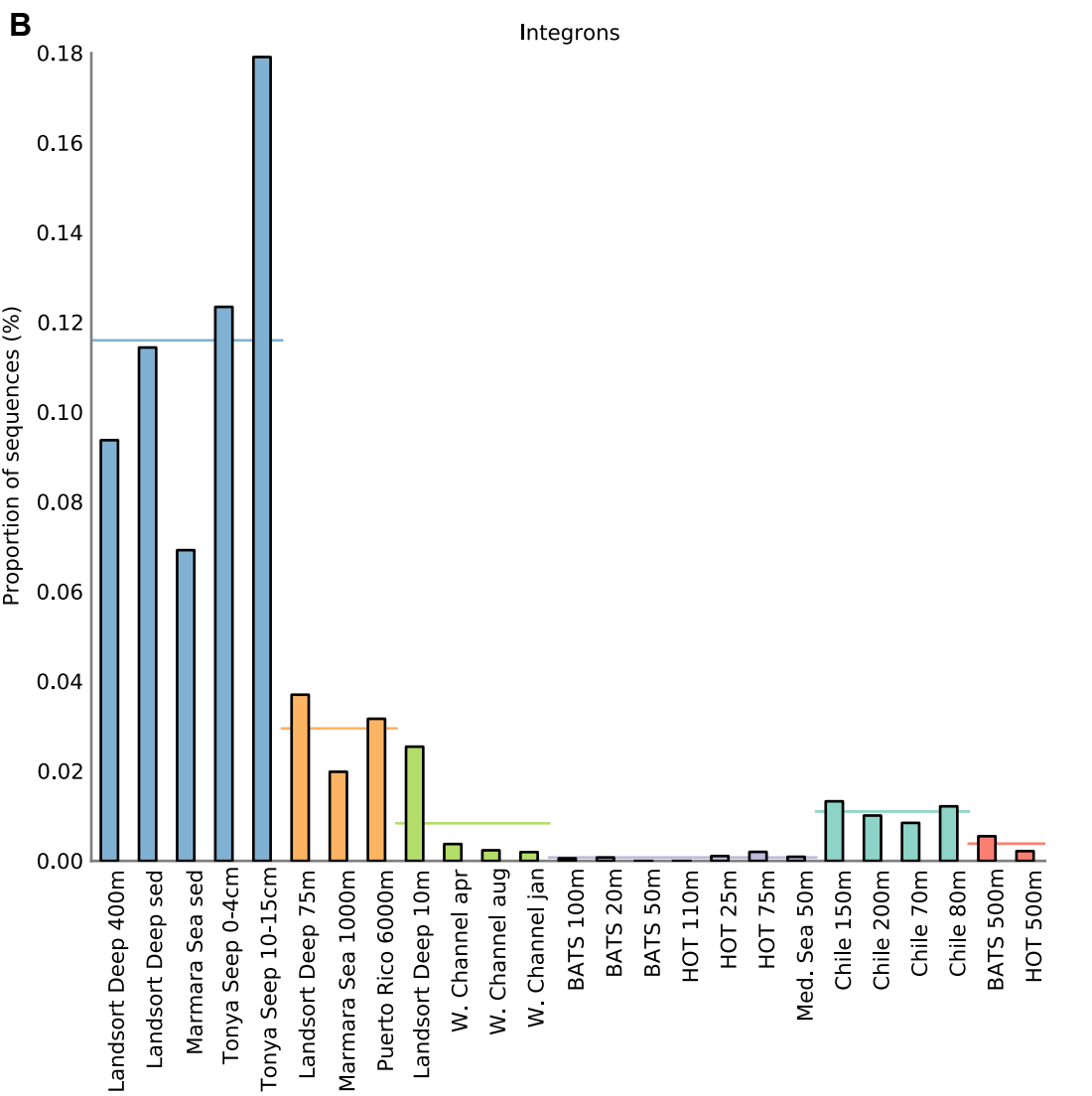

Supplement: Figure S6 — Relative abundance of sequence reads assigned to the SEED categories Cobalt zinc cadmium resistance (a) and Integrons (b), respectively, in marine metagenomes. Clustering of metagenomes is based on results of the comparative network analysis (Figure 4). Horizontal lines indicate mean value for the respective cluster. (PDF) [file pone.0074983.s006.pdf]
