# Supplementary material for: A Metagenomics Transect into the Deepest Point of the Baltic Sea Reveals Clear Stratification of Microbial Functional Capacities
Source: PLoS One. 2013 Sep 23;8(9):e74983. doi: 10.1371/journal.pone.0074983 (PMC3781128; doi:10.1371/journal.pone.0074983)
Supplement: Figure S4 — Pairwise statistical comparison of functional capacities (SEED hierarchy 1) between cluster of metagenomes. The cluster (defined by comparative network analysis) of metagenomes of Landsort Deep 400 m and sediment, Marmara Sea sediment and Tonya Seep sediments was compared to all other marine metagenomes using Welch’s t-test and Storey’s FDR (q<0.05) in STAMP. (PDF) [file pone.0074983.s004.pdf]

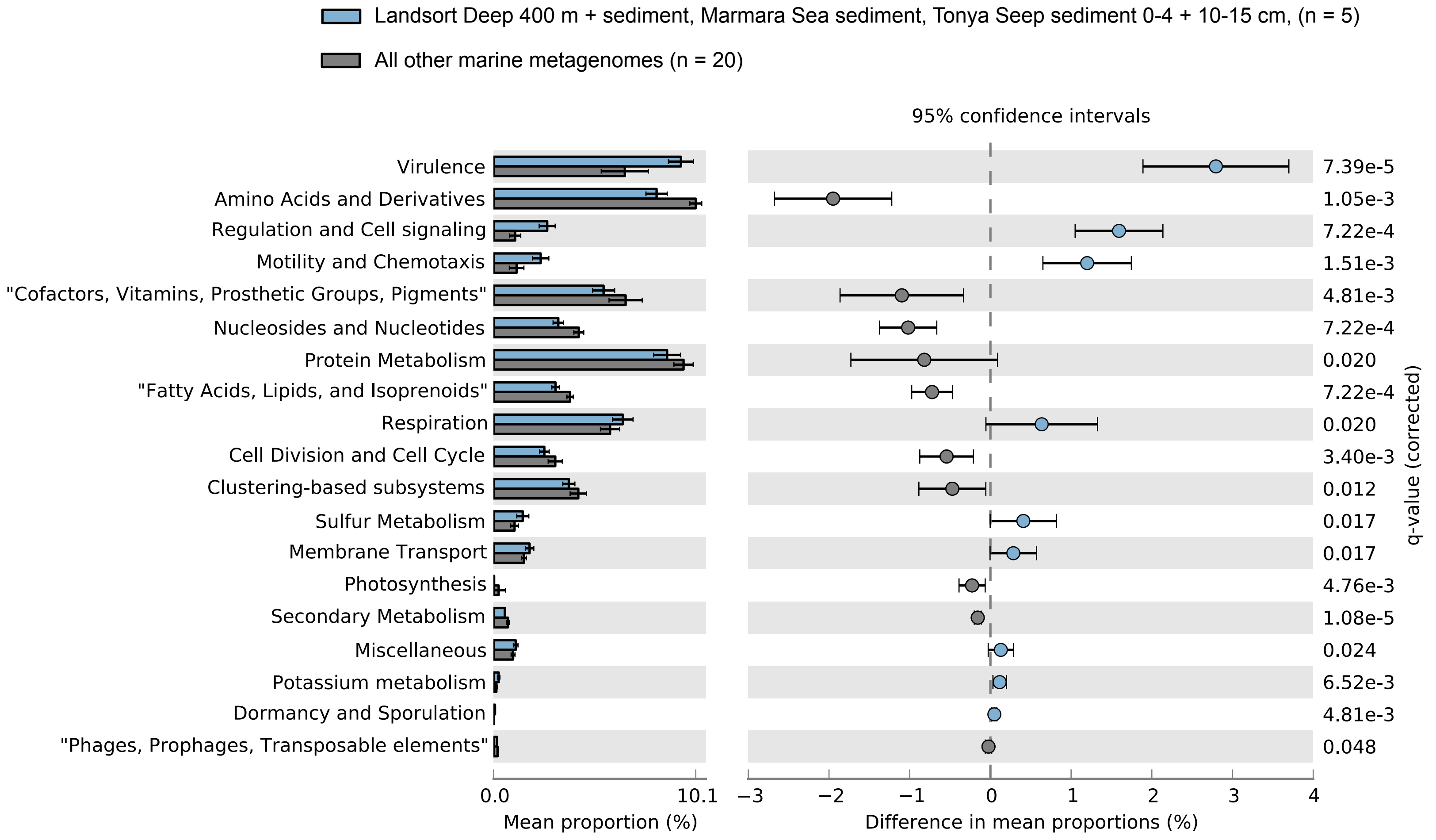

Landsort Deep 400 m + sediment, Marmara Sea sediment, Tonya Seep sediment 0-4 + 10-15 cm, (n = 5)
All other marine metagenomes (n = 20)
95% confidence intervals
Virulence
Amino Acids and Derivatives
Regulation and Cell signaling
Motility and Chemotaxis
"Cofactors, Vitamins, Prosthetic Groups, Pigments"
Nucleosides and Nucleotides
Protein Metabolism
"Fatty Acids, Lipids, and Isoprenoids"
Respiration
Cell Division and Cell Cycle
Clustering-based subsystems
Sulfur Metabolism
Membrane Transport
Photosynthesis
Secondary Metabolism
Miscellaneous
Potassium metabolism
Dormancy and Sporulation
"Phages, Prophages, Transposable elements"
7.39e-5
1.05e-3
7.22e-4
1.51e-3
4.81e-3
7.22e-4
0.020
7.22e-4
0.020
3.40e-3
0.012
0.017
0.017
4.76e-3
1.08e-5
0.024
6.52e-3
4.81e-3
0.048
q-value (corrected)
0.0
10.1
Mean proportion (%)
−3
−2
−1
0
1
2
3
4
Difference in mean proportions (%)
