# Supplementary material for: A Metagenomics Transect into the Deepest Point of the Baltic Sea Reveals Clear Stratification of Microbial Functional Capacities
Source: PLoS One. 2013 Sep 23;8(9):e74983. doi: 10.1371/journal.pone.0074983 (PMC3781128; doi:10.1371/journal.pone.0074983)
Supplement: Table S1 — Environmental parameters collected at Station BY31, Landsort Deep, Baltic Sea at the 15th of April concurrently with sampling of water and sediment for metagenomic analyses. (PDF) [file pone.0074983.s007.pdf]

Depth (m)	Temp. (°C)	Salinity (psu)	O_2 (mL/L)	H_2S (µM)	PO_4^{3-} (µM)	Total P (µM)	NO_3^-/NO_2^- (µM)	NH_4^+ (µM)	Total N (µM)	SiO_4^- (µM)	Chl a (µg/L)	pH	DOC (ug/L)
0	3.99	6.56	10.71		0.22	0.8	0.02	0.04	21.24	10.4	4.5		
5	3.96	6.52			0.22	0.8	0	0.04	21.37	10.3	3.59		
10	4.1	6.55			0.22	0.79	0	0.04	20.37	10.3	3.18		3.9
15	3.47	6.66			0.2	0.74	0	0.04	20.37	9.5	2.2		
20	3.04	6.70	9.78		0.32	0.7	0.02	0.04	18.75	10.8	1.02		
25	2.43	6.78			0.33	0.69	0	0.05	18.42	11.3			
30	2.9	6.84			0.49	0.87	0	0.04	18.46	13.6			
40	2.46	6.94	9.26		0.65	0.9	3.61	0.05	20.55	14.3	0.86		
50	2.74	7.03			0.65	0.9	3.8	0.08	20.94	13.6	0.77		
60	3.02	7.15	9.03		0.66	0.9	4.06	0.07	20.9	13.6	0.55		
70	4.24	7.92	6.55		1.29	1.5	4.88	0.08	21.35	22.3			
80	5.23	9.56	1.36		3.05	3.2	4.68	0.11	19.67	47	0.2	7.3	3.4
90	5.38	10.02	0.69		3.25	3.5	0.25	2.6	18.26	52			
100	5.49	10.27	-0.23	5.3	3.7	3.93	0.02	4.55	19.71	53.8			
125	5.6	10.53	-0.65	14.5	3.9	4.14	0.02	6.04	21.03	55.9			
150	5.7	10.69	-0.63	14.1	3.8	4.09	0.02	5.74	21.11	56.1			
175	5.74	10.71	-0.46	10.3	3.7	4	0.02	4.8	21.66	55.3			
200	5.76	10.72	-0.38	8.5	3.7	3.95	0.02	4.44	20.12	55.3			
250	5.68	10.75	-1.05	23.5	4	4.32	0.02	7.67	23.3	57.7			
300	5.7	10.81	-1.28	28.6	4.1	4.35	0.02	8.31	24.31	58			
400	5.72	10.86	-1.33	29.8	4.1		0.02	8.7		58.6		7.1	3.2
440	5.73	10.86	-1.35	30.3	4.1	4.39	0.02	9.06	24.27	58.7			
Sediment (466m)	6.3	11.14			45		3.09	416		262		7.5	43
