# Supplementary material for: A Metagenomics Transect into the Deepest Point of the Baltic Sea Reveals Clear Stratification of Microbial Functional Capacities
Source: PLoS One. 2013 Sep 23;8(9):e74983. doi: 10.1371/journal.pone.0074983 (PMC3781128; doi:10.1371/journal.pone.0074983)
Supplement: Table S2 — Summary of sequence and annotation data for the four Landsort Deep metagenomic data sets. Taxonomical and functional assignments of sequence reads were performed with MEGAN 4. (PDF) [file pone.0074983.s008.pdf]

Sample	*No. of total reads*	*No. of total reads after CD-HIT*	*Ave. read length (bp)*	*No. of reads assigned to taxa*	*% of total reads assigned to taxa*	*No. of reads assigned to SEED subsystems*	*% of total reads assigned to SEED subsystems*
10 m	185 809	157 710	334	61 437	39.0	27 291	17.3
75 m	354 912	326 263	344	165 044	50.6	74 284	22.8
400 m	434 236	407 720	352	209 545	51.4	94 614	23.2
Sediment	340 083	313 937	347	133 250	42.4	63 917	20.4
**Total**	**1 315 040**	**1 205 630**	**344**	**569 276**	**47.2**	**260 106**	**21.6**
