# Supplementary material for: A Metagenomics Transect into the Deepest Point of the Baltic Sea Reveals Clear Stratification of Microbial Functional Capacities
Source: PLoS One. 2013 Sep 23;8(9):e74983. doi: 10.1371/journal.pone.0074983 (PMC3781128; doi:10.1371/journal.pone.0074983)
Supplement: Table S3 — Ecological distances between the Landsort Deep metagenomes. Dissimilarity indices were calculated with Bray-Curtis distance metric at the taxonomic level of family. (PDF) [file pone.0074983.s009.pdf]

	10 m	75 m	400 m	Sediment 1	Sediment 2	Sediment 3
**10 m**	0.000	0.493	0.830	0.832	0.837	0.824
**75 m**	0.493	0.000	0.552	0.613	0.622	0.611
**400 m**	0.830	0.552	0.000	0.431	0.459	0.422
**Sediment 1**	0.832	0.613	0.431	0.000	0.067	0.109
**Sediment 2**	0.837	0.622	0.459	0.067	0.000	0.135
**Sediment 3**	0.824	0.611	0.422	0.109	0.135	0.000
