# Supplementary material for: A Metagenomics Transect into the Deepest Point of the Baltic Sea Reveals Clear Stratification of Microbial Functional Capacities
Source: PLoS One. 2013 Sep 23;8(9):e74983. doi: 10.1371/journal.pone.0074983 (PMC3781128; doi:10.1371/journal.pone.0074983)
Supplement: Table S4 — Correlation between environmental parameters and distribution of functional capacities and taxa, respectively. Pearson’s correlations were performed based on coordinates of SEED categories (hierarchy 2) and taxa (family rank) along the three ordination axes from correspondence analysis. (PDF) [file pone.0074983.s010.pdf]

	NH_4^+	PO_4^{3-}	$NO_3^- + NO_2^-$	SiO_4	DOC	Temperature	Salinity	O_2
CA1-SEED	0.69	0.73	0.59	0.79	0.67	0.98	0.98	-0.96
CA2-SEED	0.66	0.63	0.79	0.56	0.68	0.04	-0.10	0.06
CA3-SEED	-0.23	-0.20	0.11	-0.16	-0.24	0.17	0.19	-0.29
CA1-Taxa	0.60	0.64	0.52	0.72	0.57	0.99	1.00	-0.97
CA2-Taxa	0.79	0.75	0.76	0.68	0.80	0.10	-0.04	0.13
CA3-Taxa	0.04	0.05	0.39	0.06	0.04	0.15	0.11	-0.23
