# Supplementary material for: A Metagenomics Transect into the Deepest Point of the Baltic Sea Reveals Clear Stratification of Microbial Functional Capacities
Source: PLoS One. 2013 Sep 23;8(9):e74983. doi: 10.1371/journal.pone.0074983 (PMC3781128; doi:10.1371/journal.pone.0074983)
Supplement: Figure S5 — Comparative analysis of functional capacity of Landsort Deep communities with that of other aquatic metagenomes based on relative abundance of SEED categories (hierarchy 3). Yellow to black colour represents high and low relative abundance of SEED categories. Hierarchical clustering was performed using Kendall’s Tau distance metric and average linkage clustering. (PDF) [file pone.0074983.s005.pdf]

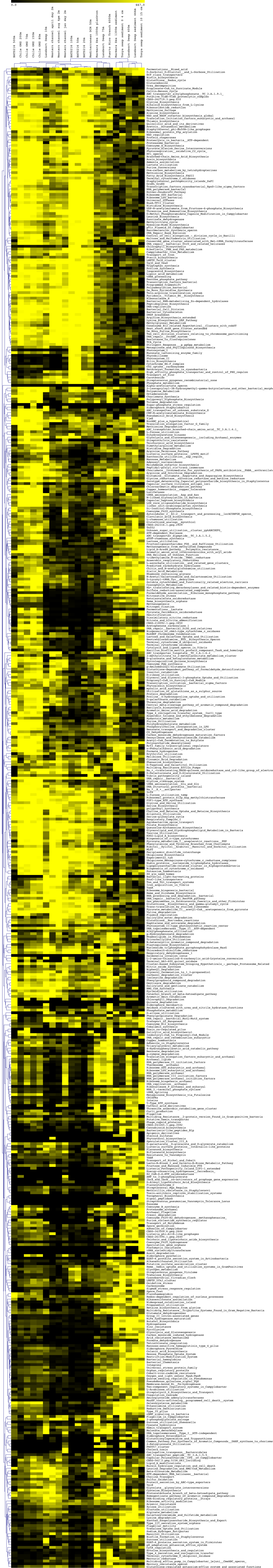
0.0
667.0
BATS216 500m
Chile OMZ 200m
Chile OMZ 70m
Chile OMZ 150m
Chile OMZ 80m
Landsort Deep 10m
Western channel april day 2m
Western channel aug 4pm 2m
Western channel jan day 2m
BATS216 100m
BATS216 50m
BATS216 20m
Mediterranean DCM 50m
Marmara Sea 1000m plankton
Landsort Deep 75m
Puerto Rico Trench 6000m
Marmara Sea 1300m sediment
Tonya seep sediment 0 4 cm
Landsort Deep 400m
Landsort Deep sediment 466m
Tonya seep sediment 10 15 cm
Fermentations__Mixed_acid
D-Sorbitol_D-Glucitol__and_L-Sorbose_Utilization
ECF_class_transporters
Biotin_biosynthesis
Glutathione__Redox_cycle
Glutaredoxins
Urea_decomposition
Propionate-CoA_to_Succinate_Module
Calvin-Benson_cycle
ABC_transporter_alkylphosphonate__TC_3.A.1.9.1_
Putative_TldE-TldD_proteolytic_complex
CBSS-262719.3.peg.410
Glycine_Biosynthesis
Alkaloid_biosynthesis_from_L-lysine
Ribonucleotide_reduction
Methionine_Salvage
Alanine_biosynthesis
NAD_and_NADP_cofactor_biosynthesis_global
Translation_initiation_factors_eukaryotic_and_archaeal
Thiamin_biosynthesis
Quinolinic_acid_and_its_derivatives
Acetoin__butanediol_metabolism
Staphylococcal_phi-Mu50B-like_prophages
Ribosomal_protein_S5p_acylation
NAD_regulation
Protein_chaperones
