# Supplementary material for: A Metagenomics Transect into the Deepest Point of the Baltic Sea Reveals Clear Stratification of Microbial Functional Capacities
Source: PLoS One. 2013 Sep 23;8(9):e74983. doi: 10.1371/journal.pone.0074983 (PMC3781128; doi:10.1371/journal.pone.0074983)
Supplement: Text S1 — Supporting material and methods. (PDF) [file pone.0074983.s012.pdf]

Text S1

Materials and Methods

Sampling

Water was sampled from three distinct zones, the surface water at 10-20 m, the mixed layer at 70-80 m and the anoxic zone at 400-410 m depth, using 5L Niskin bottles 3 for each depth, attached to a CTD line and wired down for sampling twice, pooled into 25L PVC containers and pumped trough tygon tubes(Saint-Gobain Performance Plastics Akron,USA) to GE 3 µm Polycarbonate 3 µm 47 mm prefilters (GE Healthcare, Uppsala, SWEDEN) and Sterivex GS 0.22 µm filter units (Millipore,USA) with a Gilson minipuls evolution peristaltic pump (PRETECK instruments, SWEDEN) at maximum flowrate. Sterivex filters were immediately immensed in 1.8 ml of lysis buffer (750 mM sucrose, 50 mM EDTA, 50 mM Tris-HCl, pH 8.0, 400 mM NaCl) frozen onboard and transferred on dry ice to laboratory where stored in -80°C until extraction. All equipment and solutions used were sterilised by autoclavation and sterile sealed prior to use, 25L PVC water containers were rinsed with seawater from corresponding depth.

DNA extraction

Total DNA was extracted from 200 mg triplicate sediment aliquots using the FastDNA® SPIN Kit for Soil (MP Biomedicals, Solon, OH, USA) following the manufacturer's instructions. For maximal extraction, each sediment aliquot was re-extracted twice and additional washes with 5.5 M guanidine thiocyanate (Sigma Aldrich, St. Louis, MO, USA) were included to enhance removal of humic acids. The DNA was further purified using the QIAGEN Genomic-tip 20/G (QIAGEN, Valencia, CA, USA).

DNA from water samples was extracted from the sterivex filters by the method of Somerville et al with the following modifications; 180 µl freshly made lysozyme (9 mg ml/ml in 50 µl of Tris-HCL pH 8) was added to the filters and the syringe end was sealed with parafilm and incubated for 1 h at 37°C in a rotating carousel at 27 rpm. 55µl Proteinase K (20mg/ml in 50 µl of Tris-HCL pH 8) and 200 µl sodium dodecyl sulfate (10%) was added, incubated at 55°C for 3.5 h in a rotating carousel at 27 rpm and lysate was drawn into a syringe. Filters were washed with 1 ml of lysis buffer, pooled with the lysate and divided into eppendorf tubes (total 1ml/ tube). 166 µl 3.0 M NaAcetate (pH 5.2) and 0.66 ml isopropanol was added to the mixture and incubated in -20C overnight. Samples were centrifuged at 11000 rpm 30 min, resuspended in 133µl xTE and incubated 1h 37°C, pooled in one tube and extracted twice with 500 µl of phenol-chloroform-isoamyl alcohol (25:24:1) and once with 600 µl of chloroform-isoamyl alcohol (24:1) and precipitated with 1/10 volume NaAcetate and 2.5 volume 100% EtOH in -20°C overnight then centrifuged at 11000 rpm 4°C for 30 min. and washed twice in 70% EtOH , centrifuged at 11000 rpm 4°C for 30 min. Precipitated DNA was dried in speedvac and resuspended in 50 µl of 10 mM Tris-Cl, pH 8.5. DNA quality was evaluated with NanoDrop ND-1000 (Thermo Fisher Scientific Inc., Waltham, MA, USA), Concentrations were determined by the

Qubit BR DNA assay (Invitrogen, Carlsbad, CA, USA) using the Qubit Fluorometer (Invitrogen).

Global comparative metagenomic analyses

For comparison of the Landsort Deep data with other metagenomes, a number of publicly available larger metagenomic datasets were selected. The metagenomes were retrieved from the NCBI short read archive (SRA) and the INSDC short reads archive under the following accession numbers:

Site	Sample	Accession number
HOT186	25m	SRX007372
	75m	SRX007369
	110m	SRX007370
	500m	SRX007371
BATS216	20m	SRX008032
	50m	SRX008033
	100m	SRX008035
	500m	SRX007384
Marmara Sea	1000m	SRX017437
	1300m sediment	SRX017438
Western Channel	jan day	ERR010482
	apr day	ERR010489
	aug 4pm	ERR010498
Chile OMZ station 3	70m	SRX029170
	200m	SRX029171
	80m	SRX029172
	150m	SRX029173
Mediterreanean DCM 50m	50 m	SRP002017
Waseca farm soil		AAFX01000000
Switchgrass compost		SRX014898
Tonya seep sediment	0-4cm bsf	SRX041231
	10-15cm bsf	SRX041232
Puerto Rico Trench	6000 m	SRX039182
