# Supplementary material for: A Metagenomics Transect into the Deepest Point of the Baltic Sea Reveals Clear Stratification of Microbial Functional Capacities
Source: PLoS One. 2013 Sep 23;8(9):e74983. doi: 10.1371/journal.pone.0074983 (PMC3781128; doi:10.1371/journal.pone.0074983)
Supplement: Table S5 — Species scores (coordinates) from CA for the different taxa (at the rank of family) and SEED categories (hierarchy 2), respectively. Shown are also abundances in each sample normalised to 100 000 reads per dataset. Taxa and SEED categories are ordered from low to high CA1 coordinates. (PDF) [file pone.0074983.s011.pdf]

	Species score			Abundance (normalised to 100 000 reads per dataset)			
**Taxonomical families**	CA1	CA2	CA3	10m	75m	400m	Sediment
unclassified Flavobacteria	-1.207	0.221	-0.600	2096	327	52	29
OMG group	-1.129	0.211	-0.444	4574	1106	72	173
unclassified Methylophilales	-1.107	0.133	-0.289	783	278	16	9
environmental samples 2	-1.069	0.148	-0.488	560	125	39	17
environmental samples 18	-1.064	0.074	-0.046	18	10	0	0
Comamonadaceae	-0.962	0.209	-0.521	934	174	78	92
Flavobacteriaceae	-0.921	0.090	-0.280	7756	2901	782	504
SAR11 cluster	-0.903	-0.050	0.348	7219	7684	53	13
unclassified Rhodobacterales	-0.894	0.086	-0.123	162	82	12	13
environmental samples 21	-0.894	-0.059	0.397	14	16	0	0
Rhodobacteraceae	-0.883	0.124	-0.199	3985	1723	309	412
environmental samples 20	-0.861	0.025	0.114	72	55	4	5
environmental samples 12	-0.841	-0.098	0.236	28	27	3	0
Puniceicoccaceae	-0.768	0.084	-0.164	773	373	107	110
Anaplasmataceae	-0.758	-0.153	-0.558	14	3	5	0
environmental samples 1	-0.737	-0.063	-0.307	201	79	55	13
unclassified Flavobacteriales	-0.712	0.092	-0.277	1366	522	255	232
unclassified Alphaproteobacteria	-0.601	-0.148	0.237	356	401	106	41
Microbacteriaceae	-0.580	0.032	0.290	65	74	9	19
Rickettsiaceae	-0.514	0.023	0.391	117	164	18	44
Sphingomonadaceae	-0.492	-0.146	0.234	219	264	86	46
unclassified Actinobacteria	-0.484	0.105	0.270	96	109	14	43
Sanguibacteraceae	-0.462	0.305	-0.366	20	5	4	9
environmental samples 3	-0.456	-0.396	0.969	24	122	16	0
Dermabacteraceae	-0.443	-0.412	1.570	0	22	0	0
Jonesiaceae	-0.443	-0.412	1.570	0	5	0	0
Leuconostocaceae	-0.443	-0.412	1.570	0	3	0	0
environmental samples 13	-0.443	-0.412	1.570	0	170	0	0
environmental samples 14	-0.443	-0.412	1.570	0	4	0	0
unclassified Burkholderiales miscellaneous	-0.443	-0.412	1.570	0	4	0	0
environmental samples 19	-0.443	-0.412	1.570	0	7	0	0
Halothiobacillaceae	-0.443	-0.412	1.570	0	4	0	0
SAR86 cluster	-0.443	-0.412	1.570	0	12	0	0
Flammeovirgaceae	-0.430	0.082	-0.736	14	0	6	4
Enterococcaceae	-0.402	0.112	-0.267	20	8	7	8
Pseudomonadaceae	-0.392	-0.303	1.022	212	1327	126	86
Acidimicrobiaceae	-0.384	0.082	-0.046	247	170	84	111
Acidothermaceae	-0.383	0.143	-0.162	41	21	13	19
Pseudoalteromonadaceae	-0.356	-0.111	0.325	33	51	16	14
Cellulomonadaceae	-0.349	-0.028	-0.225	14	7	7	5
Aurantimonadaceae	-0.347	-0.026	0.103	18	18	8	8
Cyclobacteriaceae	-0.342	0.165	-0.305	264	91	95	127
unclassified Gammaproteobacteria miscellaneous	-0.294	-0.020	0.085	88	88	44	45
Alteromonadales genera incertae sedis	-0.280	0.026	0.174	18	21	8	11
Xanthobacteraceae	-0.260	0.115	0.147	59	63	22	42
Burkholderiales Genera incertae sedis	-0.256	0.079	-0.049	37	27	17	22
Sphingobacteriaceae	-0.254	-0.069	-0.263	442	212	281	181
Alcaligenaceae	-0.244	-0.108	0.580	47	133	29	38
Oxalobacteraceae	-0.244	-0.154	0.331	25	45	18	14
Brucellaceae	-0.230	-0.055	0.175	18	23	11	11
Alteromonadaceae	-0.221	-0.125	0.112	101	120	73	53
Verrucomicrobiaceae	-0.217	0.188	-0.049	358	248	139	264
Brevibacteriaceae	-0.202	0.069	0.117	12	13	6	9
Hyphomonadaceae	-0.202	0.135	0.334	45	72	16	44
Caulobacteraceae	-0.201	-0.315	0.328	82	171	90	35
Intrasporangiaceae	-0.182	0.258	-0.100	22	13	8	18
Burkholderiaceae	-0.161	-0.020	0.143	211	264	142	159
Propionibacteriaceae	-0.160	0.154	-0.139	12	7	6	9
environmental samples 7	-0.150	-0.144	0.257	326	565	291	234
Pasteurellaceae	-0.139	-0.152	0.053	20	23	18	12
Actinosynnemataceae	-0.135	0.267	-0.100	10	6	4	9
Micrococcaceae	-0.122	0.187	0.048	51	47	24	49
Acetobacteraceae	-0.121	-0.058	0.382	38	84	31	39
Chitinophagaceae	-0.111	-0.062	-0.262	108	54	87	63
environmental samples 23	-0.104	-0.286	1.073	10	846	133	149
Bradyrhizobiaceae	-0.101	-0.115	0.514	122	391	127	149
Erythrobacteraceae	-0.099	0.360	0.052	31	26	9	36
Phyllobacteriaceae	-0.096	-0.008	0.241	106	174	82	106
unclassified Betaproteobacteria	-0.091	-0.007	-0.111	53	39	41	39
Glycomycetaceae	-0.081	0.239	-0.066	29	20	14	29
Idiomarinaceae	-0.076	-0.682	0.925	0	13	5	0
unclassified Verrucomicrobiales	-0.072	-0.134	0.370	100	246	112	107
Pseudonocardiaceae	-0.069	0.203	0.028	42	38	22	45
Nocardiopsaceae	-0.050	0.088	-0.068	14	11	10	13
Methylobacteriaceae	-0.049	-0.184	0.470	30	102	43	38
Rhizobiaceae	-0.049	0.086	0.144	80	105	57	90
Nitrosomonadaceae	-0.047	-0.032	0.310	38	79	36	46
Methylophilaceae	-0.031	-0.227	-0.003	16	19	20	11
Frankiaceae	-0.029	0.321	0.076	146	141	60	193
Chroococcales	-0.023	0.296	-0.123	771	447	384	862
Bifidobacteriaceae	-0.017	0.071	-0.235	14	7	11	12

Oceanospirillaceae	-0.016	-0.257	0.162	28	51	41	23
Prochlorococcaceae	-0.012	0.084	-0.187	45	26	35	41
Colwelliaceae	-0.003	-0.309	0.387	10	34	20	11
Cytophagaceae	-0.003	-0.152	-0.209	546	364	609	378
Catenulisporaceae	0.000	0.355	-0.056	25	17	11	32
Streptomycetaceae	0.004	0.231	-0.037	229	182	138	276
Nitrospinaceae	0.012	-0.747	0.769	0	19	10	0
Neisseriaceae	0.016	-0.015	0.005	24	26	24	25
Nocardioidaceae	0.016	0.339	0.001	124	101	58	170
Thermomonosporaceae	0.021	0.214	0.067	14	15	9	19
Rhodospirillaceae	0.039	-0.434	0.434	76	378	242	90
Rhodocyclaceae	0.044	-0.229	0.098	30	50	47	29
Shewanellaceae	0.052	-0.141	0.037	63	85	85	65
environmental samples 11	0.052	0.200	0.315	14	29	10	27
Reinekea	0.052	-0.777	0.699	0	10	6	0
unclassified Euryarchaeota	0.054	-0.381	0.840	0	338	117	73
Geodermatophilaceae	0.062	0.424	0.264	16	25	5	33
Enterobacteriaceae	0.072	-0.230	0.093	76	131	128	80
Nostocaceae	0.074	0.450	-0.191	179	73	80	249
environmental samples 25	0.075	-0.725	0.487	14	160	121	7
Micromonosporaceae	0.080	0.450	0.099	38	39	14	69
Kineosporiaceae	0.089	0.458	0.009	10	8	4	17
Moraxellaceae	0.092	-0.079	-0.061	28	29	36	31
Gemmatimonadaceae	0.102	0.028	0.608	33	224	63	132
Clostridiales Family XVIII Incerta	0.129	-0.199	0.794	0	51	16	19
Rhodothermaceae	0.151	-0.421	0.399	90	610	449	200
Methylococcaceae	0.163	-0.496	0.411	22	192	148	52
Actinomycetaceae	0.167	0.156	0.084	10	14	11	19
Nakamurellaceae	0.172	0.455	0.069	27	28	14	58
Alcanivoracaceae	0.203	0.066	0.061	16	24	23	31
Cenarchaeaceae	0.204	-0.889	0.431	0	53	51	0
unclassified Oceanospirillales	0.213	-0.896	0.416	0	254	251	0
Xanthomonadaceae	0.214	0.361	0.102	38	50	30	91
environmental samples 8	0.217	-0.898	0.409	0	3	3	0
Ferrimonadaceae	0.217	-0.898	0.409	0	3	3	0
Opitutaceae	0.224	-0.115	0.295	84	337	244	250
Prevotellaceae	0.234	-0.297	-0.306	49	32	99	48
Nitrosopumilaceae	0.237	-0.891	0.269	254	4219	5050	72
Streptosporangiaceae	0.239	0.423	0.055	18	20	13	44
Corynebacteriaceae	0.249	0.341	0.072	14	18	13	35
Gallionellaceae	0.255	-0.147	0.055	12	24	29	24
Beijerinckiaceae	0.259	-0.174	0.611	0	19	9	10
Carnobacteriaceae	0.266	-0.038	0.648	0	8	3	5
Spartobacteria	0.273	0.187	0.110	147	259	215	398
Beutenbergiaceae	0.274	0.224	0.050	10	14	13	25
Legionellaceae	0.279	0.189	-0.003	62	76	85	144
Lentisphaeraceae	0.289	-0.180	-0.026	96	163	247	184
Psychromonadaceae	0.295	-0.100	0.117	10	26	28	27
Leptospiraceae	0.296	0.085	-0.113	40	38	64	80
sulfur oxidizing symbionts	0.303	-0.858	0.295	0	194	232	16
Thermaceae	0.304	0.009	-0.102	22	24	41	44
Methylocystaceae	0.310	-0.311	0.484	0	16	11	8
Hyphomicrobiaceae	0.317	0.442	0.178	14	26	13	51
Brachyspiraceae	0.320	-0.170	-0.184	12	12	28	20
Hahellaceae	0.321	-0.182	0.050	10	24	32	25
Ectothiorhodospiraceae	0.321	-0.089	0.203	18	70	65	68
Acidobacteriaceae	0.325	0.029	0.034	25	45	55	67
Lactobacillaceae	0.337	0.136	-0.099	12	12	20	28
Chromatiaceae	0.338	0.031	0.189	28	93	82	111
Nocardiaceae	0.343	0.595	0.121	47	64	28	170
Porphyromonadaceae	0.343	-0.207	-0.272	28	21	67	43
Oscillatoriales	0.346	-0.017	-0.177	64	57	130	128
Staphylococcaceae	0.349	-0.312	0.425	0	9	7	5
unclassified Bacteroidetes	0.355	-0.019	-0.287	22	12	42	39
Solibacteraceae	0.358	-0.096	0.306	27	220	177	195
Bacteroidaceae	0.361	-0.123	-0.321	226	121	487	365
Bacillaceae	0.365	-0.157	0.115	67	234	282	244
Francisellaceae	0.368	-0.421	0.358	0	16	15	8
Dermacoccaceae	0.370	0.568	0.714	0	9	0	11
Thermoplasmataceae	0.380	-0.174	0.429	0	7	5	5
Vibrionaceae	0.382	-0.254	-0.083	39	72	141	91
Planctomycetaceae	0.388	0.368	0.463	447	4586	1417	6062
Promicromonosporaceae	0.396	0.157	0.526	0	12	5	12
environmental samples 16	0.418	-0.389	0.294	0	18	19	11
Bdellovibrionaceae	0.421	-0.098	-0.041	10	20	35	32
Bacteriovoracaceae	0.425	-0.215	0.004	12	35	58	43
Verrucomicrobia subdivision 3	0.433	-0.033	0.142	51	226	257	295
Methanopyraceae	0.437	-1.061	0.022	0	3	6	0
environmental samples 15	0.457	-1.075	-0.014	0	7	15	0
Rikenellaceae	0.468	-0.275	-0.362	10	6	33	19
Tsukamurellaceae	0.480	0.701	0.598	0	3	0	5
Fusobacteriaceae	0.495	-0.217	-0.198	20	28	85	59
Nannocystaceae	0.496	-0.111	0.038	16	65	103	97

Bartonellaceae	0.497	0.722	0.580	0	4	0	7
Thermomicrobiaceae	0.502	-0.183	0.244	0	20	22	20
Thiotrichaceae	0.512	-0.238	-0.113	24	57	138	96
Acidithiobacillaceae	0.523	-0.426	0.124	0	11	17	9
Halobacteriaceae	0.524	-0.028	0.071	10	50	73	82
Sphaerobacteraceae	0.526	0.161	0.333	0	62	45	87
Nitrospiraceae	0.527	-0.048	-0.002	20	67	118	123
Mariprofundaceae	0.529	-0.474	0.098	0	23	38	18
Moritellaceae	0.531	-0.277	0.166	0	11	15	11
Rubrobacteraceae	0.534	0.434	0.076	12	27	28	80
Haliangiaceae	0.537	0.036	-0.002	25	75	129	157
Lachnospiraceae	0.538	-0.059	-0.273	18	14	62	56
environmental samples 5	0.544	0.078	0.276	0	20	18	28
Streptococcaceae	0.544	0.052	0.267	0	16	15	22
Helicobacteraceae	0.558	-1.045	-0.296	32	177	701	34
unclassified Campylobacterales (Sulfurimonas gotlandica)	0.560	-1.082	-0.232	18	265	906	29
Campylobacteraceae	0.564	-0.793	-0.204	10	63	206	40
Herpetosiphonaceae	0.567	0.379	-0.039	22	33	56	127
Pelobacteraceae	0.570	0.098	0.011	14	48	81	111
Piscirickettsiaceae	0.571	-0.453	0.042	0	18	34	17
Ktedonobacteraceae	0.583	0.176	0.078	16	80	110	181
Polyangiaceae	0.584	0.099	0.029	16	68	111	154
Paenibacillaceae	0.594	-0.062	-0.122	16	37	99	96
Methanothermaceae	0.594	-1.176	-0.255	0	3	11	0
Alicyclobacillaceae	0.594	-0.053	0.153	0	18	24	27
Ferroplasmaceae	0.599	-0.224	0.084	0	3	5	4
Trueperaceae	0.604	0.252	0.250	0	16	14	30
Chlorobiaceae	0.605	-0.348	-0.273	98	160	715	398
Parachlamydiaceae	0.607	0.408	-0.117	12	12	30	67
Methanosaetaceae	0.611	0.331	0.268	0	9	7	18
Cystobacteraceae	0.614	-0.087	0.111	0	35	53	55
Ruminococcaceae	0.624	0.038	-0.217	30	34	138	152
unclassifed Acidobacteria	0.625	-0.238	-0.119	10	44	123	89
Desulfovibrionaceae	0.634	-0.124	-0.171	41	95	317	273
unclassified Proteobacteria	0.639	-0.193	0.035	0	32	61	51
Aeromonadaceae	0.652	-0.096	0.052	0	5	9	9
Methylacidiphilaceae	0.653	-0.004	0.084	0	13	21	25
Myxococcaceae	0.657	0.001	-0.048	12	66	151	170
Sulfolobaceae	0.660	-0.364	-0.058	0	6	15	9
Synergistaceae	0.661	0.124	-0.118	10	22	63	84
unclassified Bacteria	0.661	-0.162	-0.127	51	229	682	565
Chloroflexaceae	0.673	0.306	-0.049	38	97	211	400
Hydrogenophilaceae	0.676	-0.089	0.019	0	5	10	10
unclassified Cyanobacteria	0.678	0.093	0.082	0	20	32	45
Heliobacteriaceae	0.699	-0.123	-0.028	0	6	14	13
environmental samples 4	0.709	-0.079	-0.027	0	3	7	7
Dehalococcoidetes	0.711	-0.674	-0.376	18	57	392	120
Nautiliaceae	0.711	-0.853	-0.311	0	5	25	5
unclassified Planctomycetales	0.718	-0.161	-0.147	12	90	308	257
Desulfurococcaceae	0.723	-0.075	-0.046	0	6	15	15
Clostridiaceae	0.724	0.052	-0.224	61	93	474	536
Veillonellaceae	0.726	-0.143	-0.075	0	16	44	39
Halomonadaceae	0.729	-0.128	-0.074	0	4	11	10
Geobacteraceae	0.740	-0.040	-0.167	26	114	440	443
Aquificaceae	0.748	-0.397	-0.201	0	10	41	23
Coriobacteriaceae	0.755	0.092	-0.034	0	9	22	29
Coxiellaceae	0.772	0.127	-0.047	0	10	26	36
Gloeobacteria	0.775	0.070	-0.071	0	7	20	25
Thermoproteaceae	0.776	-0.060	-0.121	0	5	17	17
Methanosarcinaceae	0.777	0.204	-0.153	18	52	214	317
Deinococcaceae	0.778	0.328	0.017	0	9	18	36
Korarchaeota	0.779	-0.319	-0.219	0	3	14	9
Fibrobacteraceae	0.781	-0.344	-0.232	0	8	39	24
Syntrophaceae	0.783	0.023	-0.212	10	31	163	178
Desulfobulbaceae	0.784	-0.329	-0.379	14	20	226	136
Methanococcaceae	0.784	-0.287	-0.215	0	12	56	38
Desulfuromonadaceae	0.787	-0.103	-0.152	0	14	54	50
Methanobacteriaceae	0.789	0.082	-0.088	0	11	34	43
Waddliaceae	0.792	0.384	0.017	0	5	10	22
SAR406 cluster	0.793	-1.143	-0.539	0	8	97	7
Peptococcaceae	0.799	-0.128	-0.180	0	45	196	173
Spirochaetaceae	0.803	-0.255	-0.379	26	30	409	278
Thermococcaceae	0.807	-0.147	-0.199	0	20	95	81
Syntrophomonadaceae	0.811	-0.088	-0.183	0	18	82	77
unclassified Epsilonproteobacteria	0.813	-0.076	-0.181	0	31	141	135
Oscillochloridaceae	0.814	0.248	-0.065	0	14	41	68
Hydrogenothermaceae	0.817	-0.342	-0.284	0	6	39	24
environmental samples 24	0.821	0.155	-0.110	0	7	25	35
Dictyoglomaceae	0.823	-0.032	-0.180	0	9	42	43
Halanaerobiaceae	0.826	-0.329	-0.293	0	10	70	44
Thermoanaerobacteraceae	0.831	-0.123	-0.226	0	22	125	110
Victivallaceae	0.832	-0.345	-0.307	0	54	407	249
Deferribacteraceae	0.832	-0.389	-0.324	0	11	88	50

Desulfohalobiaceae	0.841	-0.116	-0.238	0	27	167	148
Methanomicrobiaceae	0.845	-0.022	-0.209	0	6	34	35
Elusimicrobiaceae	0.845	-0.341	-0.325	0	3	26	16
Desulfarculaceae	0.847	-0.235	-0.289	0	16	123	90
Syntrophobacteraceae	0.847	-0.044	-0.319	10	16	206	197
unclassified Methanomicrobiales	0.850	-0.009	-0.212	0	7	41	43
Eubacteriaceae	0.856	0.083	-0.188	0	6	33	40
Archaeoglobaceae	0.858	-0.353	-0.349	0	8	83	50
Methanospirillaceae	0.860	-0.201	-0.298	0	3	26	20
unclassified Deltaproteobacteria (deltaproteobacterium NaphS2)	0.866	-0.675	-0.507	53	193	5469	1848
Desulfobacteraceae	0.869	-0.037	-0.327	126	217	3425	3306
Thermotogaceae	0.871	0.030	-0.230	0	15	106	117
Methanocellaceae	0.874	-0.107	-0.283	0	3	27	24
Thermoanaerobacterales Family III Incertae Sedis	0.875	-0.031	-0.257	0	7	57	57
unclassified Bacteroidales	0.877	-1.385	-0.753	0	0	3	0
environmental samples 6	0.877	-1.385	-0.753	0	0	6	0
Peptostreptococcaceae	0.877	-1.385	-0.753	0	0	3	0
environmental samples 9	0.877	-1.385	-0.753	0	0	8	0
Thermodesulfobiaceae	0.877	-1.385	-0.753	0	0	4	0
Acidaminococcaceae	0.877	-1.385	-0.753	0	0	6	0
environmental samples 10	0.877	-1.385	-0.753	0	0	3	0
environmental samples 17	0.877	-1.385	-0.753	0	0	4	0
unclassified Alteromonadales	0.877	-1.385	-0.753	0	0	3	0
unclassified Thiotrichales	0.877	-1.385	-0.753	0	0	4	0
unclassified Thermotogales	0.884	0.061	-0.238	0	5	40	46
Erysipelotrichaceae	0.885	0.219	-0.181	0	5	31	46
Mycobacteriaceae	0.891	1.217	0.032	230	279	117	6315
unclassified Clostridiales	0.901	-0.186	-0.352	0	3	50	39
Nanoarchaeota	0.902	-0.950	-0.632	0	0	32	6
Desulfomicrobiaceae	0.906	0.206	-0.218	0	4	35	50
Conexibacteraceae	0.913	1.236	0.034	102	142	57	3558
Thermofilaceae	0.933	-0.401	-0.479	0	0	9	5
environmental samples 22	0.940	-0.283	-0.446	0	0	6	4
Methanocaldococcaceae	0.940	-0.283	-0.446	0	0	21	14
Listeriaceae	0.947	-0.161	-0.412	0	0	5	4
Clostridiales Family XI Incertae Sedis	0.947	-0.161	-0.412	0	0	15	12
Bacillales Family XII Incertae Sedis	0.948	-0.133	-0.404	0	0	6	5
Halobacteroidaceae	0.953	-0.055	-0.383	0	0	30	28
Mycoplasmataceae	0.956	-0.008	-0.369	0	0	5	5
Natranaerobiaceae	0.963	0.125	-0.332	0	0	14	17
Parvularculaceae	0.964	0.145	-0.327	0	0	4	5
Aerococcaceae	0.967	0.189	-0.315	0	0	6	8
Methanocorpusculaceae	0.967	0.189	-0.315	0	0	6	8
Cardiobacteriaceae	0.975	0.336	-0.274	0	0	3	5
Chlamydiaceae	0.977	0.368	-0.265	0	0	4	7
Acholeplasmataceae	0.992	0.635	-0.191	0	0	8	22
Gordoniaceae	1.034	1.369	0.014	0	0	0	24
Segniliparaceae	1.034	1.369	0.014	0	0	0	6

SEED Hierarchy 2	Species scores CA1	CA2	CA3	Abundance (normalised to 100 000 reads per dataset) 10m	75m	400m	Sediment
Agrobacterium opine transport	-0.889	0.123	-0.433	18	5	2	1
L 2 amino thiazoline 4 carboxylic acid Lcysteine conversion	-0.857	0.067	0.582	2	3	0	0
Amidase clustered with urea and nitrile hydratase functions	-0.724	0.326	-0.133	4	2	0	1
DNA binding regulatory proteins strays	-0.595	0.069	-0.023	93	62	21	19
Alkylphosphonate utilization	-0.580	0.100	-0.205	76	35	17	17
p cymene degradation	-0.580	-0.094	1.658	0	4	0	0
Cyanate hydrolysis	-0.552	0.014	-0.090	10	6	3	2
Dimethylarginine metabolism	-0.519	0.152	-0.227	37	16	8	11
Biogenesis of cytochrome c oxidases	-0.513	-0.030	-0.006	169	122	61	35
Phosphonate metabolism	-0.495	0.014	-0.044	24	16	8	6
Aromatic Amin Catabolism	-0.479	0.141	-0.085	47	28	11	16
Gentisare degradation	-0.461	0.074	0.110	82	73	24	28
Salicylate and gentisate catabolism	-0.433	0.040	0.122	86	80	30	30
Pyrimidines	-0.428	0.037	-0.120	120	69	43	38
YaaA	-0.371	-0.067	-0.120	20	12	10	6
Plant Alkaloids	-0.343	-0.294	0.150	35	40	30	6
Allantoin Utilization	-0.317	0.146	0.377	16	25	5	11
Iron Scavenging Mechanisms	-0.314	0.015	-0.136	1472	841	683	606
Ton and Tol transport systems	-0.300	-0.019	-0.146	1554	877	792	624
Social motility and nonflagellar swimming in bacteria	-0.298	0.323	-0.031	6	4	1	4
Metabolism of central aromatic intermediates	-0.289	0.079	0.170	350	383	151	203
Lysine threonine methionine and cysteine	-0.278	0.022	0.027	12	10	6	6
Benzoate transport and degradation cluster	-0.257	-0.011	0.152	110	122	64	60
Adenosyl nucleosidases	-0.254	0.153	-0.154	51	27	19	29
Pyridoxine	-0.238	0.015	-0.007	143	113	78	77
Proline and 4 hydroxyproline	-0.218	0.044	0.123	213	224	117	137
Histidine Metabolism	-0.218	-0.011	0.034	483	426	293	271
Detoxification 1	-0.214	-0.041	0.052	113	105	74	62
Alanine serine and glycine	-0.203	-0.010	-0.015	749	596	460	424
Transport of Manganese	-0.199	0.144	-0.201	104	49	44	64
Organic acids	-0.198	0.019	0.017	374	318	220	228
YgjD and YeaZ	-0.190	-0.033	-0.064	113	81	73	62
Thioredoxin disulfide reductase	-0.186	0.029	0.035	98	87	58	63
LMPTP YwlE cluster	-0.180	-0.008	-0.052	143	105	90	84
Biologically active compounds in metazoan cell defence and differentiation	-0.177	0.056	0.061	49	46	28	34
Secretion	-0.175	-0.065	0.076	75	76	56	45
Stringent Response p ppGpp metabolism	-0.169	0.000	-0.095	130	86	81	77
Choline Transport	-0.152	0.364	-0.362	8	2	2	6
ATP synthases	-0.151	-0.104	-0.077	460	332	360	250
YcfH	-0.148	-0.157	-0.115	51	34	43	25
Plant Hormones	-0.145	-0.022	0.107	164	179	121	116
Clustering based subsystems 1	-0.144	0.003	-0.011	169	139	113	113
Biogenesis of c type cytochromes	-0.134	-0.005	0.061	259	255	185	186
UDP N acetylmuramate from Fructose 6 phosphate Biosynthesis	-0.131	-0.003	0.022	195	175	137	137
Isoprenoids	-0.123	0.043	-0.006	654	543	420	485
carbazol degradation cluster	-0.117	0.303	0.111	26	26	8	27
CO2 fixation	-0.116	0.084	0.107	652	700	404	561
Purines	-0.115	-0.020	0.004	1139	996	847	807
Branched chain amino acids	-0.108	0.093	-0.003	1180	978	701	956
Carbon monoxide dehydrogenase maturation factors	-0.106	0.158	0.141	36	41	19	35
Thiamin biosynthesis	-0.102	-0.024	0.068	227	234	180	174
Bacterial cytostatics differentiation factors and antibiotics	-0.100	0.078	0.011	67	58	42	55
Heat shock dnaK gene cluster extended	-0.097	-0.041	-0.047	653	511	511	454
Hydantoin metabolism	-0.096	0.000	0.139	122	148	97	104
De Novo Pyrimidine Synthesis	-0.091	0.022	0.036	302	285	220	244
Quinone cofactors	-0.087	0.014	0.120	371	431	289	323
Staphylococcal phi Mu50B like prophages	-0.087	0.000	0.129	67	80	54	58
Control of Macromolecuar Synthesis	-0.084	-0.022	-0.002	509	447	402	387
NAD and NADP	-0.084	0.008	0.043	332	322	253	271
NusA TFII Cluster	-0.083	-0.075	0.020	239	226	211	174
RNA processing and modification	-0.080	-0.028	0.010	1454	1320	1179	1118
Lysine threonine methionine and cysteine 1	-0.080	0.000	0.041	1739	1690	1356	1417
Purine Utilization	-0.077	-0.099	0.037	173	173	163	126
Coenzyme A	-0.077	0.014	0.058	239	241	184	202
Central carbohydrate metabolism	-0.075	0.004	0.006	2188	1952	1681	1760
Uni Sym and Antiporters	-0.074	0.113	-0.030	78	61	47	68
X urea cycle polyamines	-0.074	0.051	0.011	807	716	565	686
Osmotic stress	-0.072	0.184	0.073	291	287	152	295
Oxidative stress	-0.067	0.029	0.082	485	520	374	436
ammonia assimilation	-0.066	0.074	0.030	139	129	95	125
Fatty acids	-0.065	0.022	0.041	704	684	541	609
DNA structural proteins bacterial	-0.065	-0.169	-0.055	44	36	45	28
Protein biosynthesis	-0.060	-0.078	-0.012	3096	2744	2821	2329
DNA replication	-0.058	-0.033	-0.011	1991	1741	1683	1584
Phospholipids	-0.056	0.038	0.019	548	505	411	484
Peptidoglycan Biosynthesis	-0.055	0.009	-0.040	977	782	756	800
Ammonia assimilation	-0.053	0.024	0.098	563	635	456	530
Lipoic acid	-0.047	0.130	-0.004	76	64	47	73
CBSS 562 2 peg 5158 SK3 including	-0.046	-0.037	0.201	2	3	2	2
Transport of Zinc	-0.044	-0.109	0.086	103	120	109	85
Biotin	-0.043	-0.002	0.042	141	141	119	126
Sex pheromones in Enterococcus faecalis and other Firmicutes	-0.042	0.084	-0.154	118	67	77	98
Bacterial Cell Division	-0.042	-0.015	-0.023	1039	884	870	869
Folate and pterines	-0.041	-0.038	-0.034	635	529	552	515
Detoxification	-0.035	-0.008	0.055	24	25	21	22

proteosome related	-0.033	0.145	-0.029	110	87	67	108
DNA repair	-0.033	-0.049	-0.033	1903	1606	1715	1558
Ribonucleotide reduction	-0.027	-0.119	0.106	205	256	232	179
Hfl operon	-0.024	-0.060	0.146	94	127	99	92
Pathogenicity islands	-0.024	0.019	-0.022	166	141	135	150
Bacterial RNA metabolizing Zn dependent hydrolases	-0.018	0.012	-0.035	398	329	330	358
Glutathione regulated potassium efflux system and associated functions	-0.018	-0.159	-0.025	110	100	123	83
Transcription	-0.017	-0.052	0.006	1363	1291	1303	1196
Protein folding	-0.016	-0.058	0.004	690	652	667	603
Peripheral pathways for catabolism of aromatic compounds	-0.012	0.058	0.101	408	474	337	440
Phosphate metabolism	-0.009	0.034	-0.004	762	683	626	735
ZZ gjo need homes	-0.005	0.060	0.019	494	466	393	502
pH adaptation potassium efflux system	-0.001	0.238	-0.179	2	1	1	2
Cell cycle in Prokaryota	-0.001	-0.026	-0.018	1065	946	989	978
Invasion and intracellular resistance	0.005	-0.008	0.086	227	265	221	238
Aromatic amino acids and derivatives	0.007	0.049	0.073	206	227	178	225
Gram Negative cell wall components	0.013	-0.016	-0.019	714	635	668	684
Tetrapyrroles	0.017	-0.025	0.022	718	720	708	716
One carbon Metabolism	0.028	0.024	0.051	714	764	669	787
Potassium homeostasis	0.028	-0.010	-0.016	254	230	243	255
DNA recombination	0.031	-0.033	-0.108	114	80	108	104
Riboflavin FMN FAD	0.032	0.074	0.035	129	130	109	147
YjeE	0.035	-0.189	-0.043	87	79	111	72
Protein degradation	0.037	0.015	0.033	713	735	685	783
Muconate lactonizing enzyme family	0.045	0.197	0.009	20	18	13	25
Sulfur oxidation	0.051	-0.033	-0.001	92	89	97	97
Electron donating reactions	0.053	-0.049	0.000	1251	1227	1367	1311
LMPTP YfkJ cluster	0.073	0.061	-0.010	26	24	24	31
Organic sulfur assimilation	0.080	0.173	0.208	251	403	213	419
Capsular and extracellular polysacchrides	0.087	0.040	-0.035	692	604	672	818
Conserved gene cluster associated with Met tRNA formyltransferase	0.088	0.084	-0.033	454	392	407	556
High affinity phosphate transporter and control of PHO regulon	0.088	0.081	-0.041	224	189	201	272
Electron accepting reactions	0.101	-0.052	0.037	499	569	626	616
Polyhydroxybutyrate metabolism	0.102	0.159	0.059	80	88	67	117
Detection	0.106	-0.061	0.039	45	52	58	56
Resistance to antibiotics and toxic compounds	0.107	-0.027	-0.004	1604	1607	1878	1954
Aromatic amino acids and derivatives 1	0.110	0.203	0.143	16	22	13	27
Formate hydrogenase	0.113	0.000	-0.004	168	167	190	212
Protein processing and modification	0.113	-0.006	-0.046	332	292	368	400
Flagellar motility in Prokaryota	0.120	-0.025	-0.024	738	704	873	913
Monosaccharides	0.133	0.090	-0.106	1189	837	1107	1510
Biosynthesis of phenylpropanoids	0.134	0.179	-0.135	26	16	20	34
Cell wall of Mycobacteria	0.135	0.159	-0.041	542	457	460	770
Coenzyme F420	0.137	0.019	0.113	28	40	36	44
ABC transporters	0.141	0.012	0.065	201	251	252	299
Fermentation	0.156	-0.025	0.009	465	508	613	656
Type III secretion system extended	0.163	0.128	0.808	0	12	3	7
YbbK	0.163	0.075	0.080	27	35	32	45
Nitric oxide synthase	0.177	0.341	0.172	51	77	34	112
Adhesion	0.178	-0.049	0.119	129	206	211	221
Transport of Molybdenum	0.184	-0.004	0.105	4	6	6	7
Glycoside hydrolases	0.187	0.047	-0.085	98	78	112	140
Catabolism of an unknown compound	0.196	0.457	0.084	16	18	7	34
Anaerobic degradation of aromatic compounds	0.200	-0.400	0.388	2	13	13	6
Universal stress protein family	0.214	0.107	0.012	22	24	26	39
Regulation of virulence	0.219	0.121	-0.088	131	102	140	210
Conjugative transposon Bacteroidales	0.219	-0.980	0.405	0	2	3	0
Cytochrome B6 F complex	0.225	0.047	0.095	2	3	3	4
Bacteriophage structural proteins	0.234	0.117	0.349	2	7	4	7
Selenoproteins	0.236	-0.089	-0.005	123	145	215	204
Soluble cytochromes and functionally related electron carriers	0.240	-0.071	-0.076	166	151	262	255
Programmed Cell Death and Toxin antitoxin Systems	0.245	0.171	-0.061	40	34	42	72
Tn552	0.276	0.310	-0.054	18	15	15	37
Sugar alcohols	0.282	0.182	-0.052	342	308	387	683
Triacylglycerols	0.307	-0.027	0.139	8	17	19	22
Di and oligosaccharides	0.316	0.163	-0.119	444	321	534	878
Electron transport and photophosphorylation	0.324	0.230	-0.028	6	6	7	14
Archease	0.331	-0.276	-0.010	2	3	6	4
Antibacterial peptides	0.335	0.065	0.576	0	6	3	5
Biogenesis of cbb3 type cytochrome c oxidases	0.336	-0.518	0.206	6	33	53	21
Coenzyme B	0.348	-0.138	0.498	0	4	3	3
Denitrification	0.349	-0.427	0.223	12	69	102	51
Gram Positive cell wall components	0.365	0.079	-0.094	67	59	109	151
Aminosugars	0.383	0.072	-0.069	119	119	212	292
Cresol degradation	0.391	0.143	0.529	0	2	1	2
Two component regulatory systems in Campylobacter	0.397	0.002	-0.047	161	189	349	419
Type III secretion system related	0.402	0.215	0.092	10	18	18	36
Nitrate and nitrite ammonification	0.409	-0.305	0.185	22	114	172	117
Transport of Nickel and Cobalt	0.422	-0.016	0.017	14	23	38	45
Type III Type IV Type VI ESAT secretion systems	0.425	-0.030	0.037	219	402	644	746
Sugar Phosphotransferase Systems PTS	0.428	0.098	0.023	16	25	36	54
Bacterial Chemotaxis	0.440	-0.056	-0.016	125	190	363	396
Pseudomonas quinolone signal PQS	0.441	0.030	-0.151	4	3	8	10
cAMP signaling in bacteria	0.443	0.108	0.059	113	212	282	437
CBSS 261594 1 peg 2640	0.455	0.238	0.072	6	11	12	25
ECF class transporters	0.456	0.275	-0.035	26	29	40	87
Carbohydrates 1	0.459	0.181	-0.208	4	2	6	10

Restriction Modification System	0.461	-0.219	-0.003	102	202	444	358
PA0057 cluster	0.478	0.704	0.591	0	2	0	3
Luciferases	0.478	0.704	0.591	0	2	0	3
Flavohaemoglobin	0.480	0.044	0.390	0	5	4	6
Orphan regulatory proteins	0.487	0.005	0.096	38	109	159	203
Sporulation gene orphans	0.514	-0.127	-0.065	8	12	31	30
CytR regulation	0.525	-0.005	0.321	0	3	3	4
Plant Octadecanoids	0.532	0.562	0.051	2	3	2	10
Group II intron associated genes	0.558	-0.008	0.017	31	75	147	182
Oxygen and light sensor PpaA PpsR	0.563	0.043	-0.021	6	11	23	31
Quorum sensing and biofilm formation	0.564	-0.127	-0.110	10	13	42	41
Putative hemin transporter	0.566	-0.148	0.227	0	7	10	10
Sodium Ion Coupled Energetics	0.573	-0.617	-0.007	2	15	49	20
Bacterial hemoglobins	0.576	-0.115	0.013	20	59	132	135
Reductive Dechlorination	0.578	-0.177	-0.102	2	3	10	9
Polysaccharides	0.590	0.104	0.030	38	98	173	266
Galactosylceramide and Sulfatide metabolism	0.594	-0.132	-0.129	16	20	73	71
Listeria phi A118 like prophages	0.596	0.035	-0.027	2	4	9	12
CRISPRs	0.602	-0.102	-0.001	8	24	57	60
Light harvesting complexes	0.615	0.423	-0.011	2	3	4	12
Dissimilatory nitrite reductase	0.618	-0.650	0.012	0	6	20	8
Spore germination	0.619	0.102	0.236	0	7	8	13
SigmaB stress responce regulation	0.647	-0.029	-0.055	29	64	180	215
Putative sulfate assimilation cluster	0.658	-0.153	0.112	0	1	2	2
Sulfate reduction associated complexes	0.665	-0.337	-0.074	6	23	88	63
Streptococcus pyogenes Virulome	0.673	0.264	0.088	2	10	14	30
CBSS 214092 1 peg 3450	0.674	-0.389	0.022	0	1	3	2
Spore Coat	0.744	0.409	0.174	0	2	2	6
Nitrosative stress	0.751	-0.211	-0.020	0	14	47	43
Lipid derived mediators	0.775	-0.064	-0.006	0	2	6	7
Tetrathionate respiration	0.790	-0.331	-0.105	0	4	21	16
Proteolytic pathway	0.836	0.583	0.112	0	3	3	13
Lacto N Biose I and Galacto N Biose Metabolic Pathway	0.839	0.445	0.068	0	2	3	9
Toxins and superantigens	0.852	0.425	0.045	0	7	12	34
AMP to 3 phosphoglycerate	0.860	-0.869	-0.352	0	0	3	1
Acid stress	0.870	-0.018	-0.110	0	3	16	20
Nitrogen fixation	0.899	-0.114	-0.174	0	7	65	70
Carbon monoxide induced hydrogenase	0.920	-0.238	-0.238	0	4	89	80
Bacillus biofilm matrix protein component TasA and homologs	0.924	-0.448	-0.305	0	0	6	4
Siderophore Enterobactin	0.962	1.070	0.103	0	1	0	7
IbrA and IbrB co activators of prophage gene expression	0.967	-0.167	-0.274	0	0	2	2
Flavocytochrome C	0.967	-0.167	-0.274	0	0	1	1
Sporulation Cluster III A	1.183	1.236	-0.119	0	0	0	1
